# Hyperbaric Oxygen Therapy Can Diminish Fibromyalgia Syndrome – Prospective Clinical Trial

**DOI:** 10.1371/journal.pone.0127012

**Published:** 2015-05-26

**Authors:** Shai Efrati, Haim Golan, Yair Bechor, Yifat Faran, Shir Daphna-Tekoah, Gal Sekler, Gregori Fishlev, Jacob N. Ablin, Jacob Bergan, Olga Volkov, Mony Friedman, Eshel Ben-Jacob, Dan Buskila

**Affiliations:** 1 Research and Development Unit, Assaf Harofeh Medical Center, Zerifin, Israel; 2 The Institute of Hyperbaric Medicine, Assaf Harofeh Medical Center, Zerifin, Israel; 3 Sackler School of Medicine, Tel-Aviv University, Tel-Aviv, Israel; 4 Sagol School of Neuroscience, Tel-Aviv University, Tel-Aviv, Israel; 5 Nuclear Medicine institute, Assaf Harofeh Medical Center, Zerifin, Israel; 6 School of Social Work, Ashkelon Academic College, Ashkelon, Israel; 7 Social Work Department, Kaplan Medical Center, Rehovot, Israel; 8 School of Physics and Astronomy, The Raymond and Beverly Sackler Faculty of Exact Sciences, Tel-Aviv University, Tel-Aviv, Israel; 9 Institute of Rheumatology, Tel Aviv Sourasky medical center Israel, Tel- Aviv, Israel; 10 Center for Theoretical Biological Physics, Rice University, Houston, Texas, United States of America; 11 Department of Medicine H, Soroka Medical Center, BGU University of the Negev, Beer Sheva, Israel; University of Sevilla, SPAIN

## Abstract

**Background:**

Fibromyalgia Syndrome (FMS) is a persistent and debilitating disorder estimated to impair the quality of life of 2–4% of the population, with 9:1 female-to-male incidence ratio. FMS is an important representative example of central nervous system sensitization and is associated with abnormal brain activity. Key symptoms include chronic widespread pain, allodynia and diffuse tenderness, along with fatigue and sleep disturbance. The syndrome is still elusive and refractory. The goal of this study was to evaluate the effect of hyperbaric oxygen therapy (HBOT) on symptoms and brain activity in FMS.

**Methods and Findings:**

A prospective, active control, crossover clinical trial. Patients were randomly assigned to treated and crossover groups: The treated group patients were evaluated at baseline and after HBOT. Patients in the crossover-control group were evaluated three times: baseline, after a control period of no treatment, and after HBOT. Evaluations consisted of physical examination, including tender point count and pain threshold, extensive evaluation of quality of life, and single photon emission computed tomography (SPECT) imaging for evaluation of brain activity. The HBOT protocol comprised 40 sessions, 5 days/week, 90 minutes, 100% oxygen at 2ATA. Sixty female patients were included, aged 21–67 years and diagnosed with FMS at least 2 years earlier. HBOT in both groups led to significant amelioration of all FMS symptoms, with significant improvement in life quality. Analysis of SPECT imaging revealed rectification of the abnormal brain activity: decrease of the hyperactivity mainly in the posterior region and elevation of the reduced activity mainly in frontal areas. No improvement in any of the parameters was observed following the control period.

**Conclusions:**

The study provides evidence that HBOT can improve the symptoms and life quality of FMS patients. Moreover, it shows that HBOT can induce neuroplasticity and significantly rectify abnormal brain activity in pain related areas of FMS patients.

**Trial Registration:**

ClinicalTrials.gov NCT01827683

## Introduction

Fibromyalgia Syndrome (FMS) is a persistent and debilitating disorder estimated to impair the quality of life of 2–4% of the population, with 9:1 female-to-male incidence ratio. FMS is the second most common disorder, after osteoarthritis, observed by rheumatologists [1]. The defining symptoms of FMS include chronic widespread pain, intense pain in response to tactile pressure (allodynia), prolonged muscle spasms, weakness in the limbs, nerve pain, muscle twitching, palpitations and diffuse tenderness, along with fatigue, sleep disturbance and cognitive impairments. These impairments include problems with short- and long- term memory, short-term memory consolidation, impaired speed of information processing, reduced attention span and limited multi-tasking performance. FMS is a persistent disorder with symptoms that have a devastating effect on people's lives, including limited ability to engage in everyday activities, limited ability to maintain outside work and difficulties to maintain normal relationships with family, friends and employers [2]. These limitations can lead to the occurrence of anxiety and depression in many FMS patients.

### Challenging syndrome

FMS is not completely understood, in part because there is no evidence of a single event that “causes” fibromyalgia. Rather, many physical and/or emotional stressors may trigger or aggravate symptoms. Those have included certain infections, such as a viral illness or Lyme disease, as well as emotional or physical trauma [3, 4]

Establishing proper diagnostic criteria is also a challenge [5, 6]. The American College of Rheumatology (ACR) introduced the first fibromyalgia classification in 1990 [7]. Over time, those criteria invoked both conceptual and practical objections [6]. For example, many physicians did not know how to evaluate the tender points [6]. Another reservation had to do with the fact that important features such as fatigue and cognitive symptoms were not included in the 1990 criteria. Some questioned the validity of defining fibromyalgia as a unique syndrome because of the overlap between its symptoms and those of other conditions such as chronic fatigue syndrome [8]. To resolve the difficulties associated with the classification and diagnosis of FMS, Wolfe et al. [6] proposed new, simple practical criteria that do not require tender point examination and still classify correctly almost 90% of the cases diagnosed by the 1990 ACR classification criteria.

As with many other syndromes, there is no efficient cure for FMS and no agreed upon treatment – the suggested treatment depends on the classification of choice. Those who regard FMS as a neurological disorder advocate pharmacotherapy. All current treatments, such as prescribed medications, aerobic exercises and cognitive behavioral therapies, consist of symptom management [1, 9, 10]. Integrated programs based on these treatments have been shown to alleviate pain and some other symptoms but with limited effectiveness [10].

### Association with changes in brain activity

The level of pain sensation is determined by the relevant sensors recording at the location of the pain and by the processing of that information in the brain. Comparison between SPECT imaging of FMS patients and healthy subjects revealed elevated activity in the somatosensory cortex and reduced activity in the frontal, cingulate, medial temporal and cerebellar cortices [11, 12]. These results are in agreement with earlier studies based on fMRI imaging [13]. Other fMRI studies found that depressive symptoms were associated with the pain response in areas of the brain that participate in interpretation and assignment of the pain sensation, but not in areas involved in sensory processing of the input signal [14]. These findings might indicate that the amplified pain sensation in FMS patients is largely associated with higher level processing of information in the brain. However, there is an ongoing controversy, in which many rheumatologists take the opposite stand on this issue. As we explain in the discussion, our findings—that the pain amelioration in those patients who responded to the HBOT treatments goes hand-in-hand with changes in brain activity—provide important validation to the idea that in many of FMS patients the syndrome is associated with abnormal pain processing in the brain. This is opposed to the stand shared by other rheumatologists, according to which FMS is a sort of peripheral small fiber inflammation [15]. It is likely that the latter is the cause of FMS in some patients. However, a claim that it is the only cause stands in contradiction to a wide body of literature. For example, it fails to explain why FMS appears in many patients following a traumatic brain injury.

Studies of brain metabolism using single-voxel magnetic resonance spectroscopy (1H-MRS) found abnormalities within the hippocampal complex in patients with fibromyalgia [16, 17]. Since the hippocampus plays crucial roles in maintenance of cognitive functions, sleep regulation and pain perception, it was suggested to associate the hippocampal metabolic dysfunction with these symptoms in FMS patients.

The evidence suggests that the pain in fibromyalgia results primarily from abnormalities in pain processing pathways, which may be described as the “volume” of the neurons set too high, and these hyper-excitability of pain processing pathways and under-activity of inhibitory pain pathways in the brain result in pain experience in the affected individual. Since some of the neuro-chemical abnormalities that occur in fibromyalgia can also regulate mood, sleep and energy, it might explain why mood, sleep and fatigue problems are commonly co-morbid with fibromyalgia.

### Looking for a solution – Hyperbaric oxygen therapy (HBOT)

Clearly, new methods should be examined in order to provide sustained relief to FMS patients. Our study was motivated by the idea that hyperbaric oxygen therapy (HBOT) can rectify abnormal brain function underlying the symptoms of FMS patients. The hypothesis is based on new trials demonstrating that HBOT can induce neuroplasticity that leads to repair of chronically impaired brain functions and improved quality of life in post-stroke patients and mild Traumatic Brain Injury (mTBI) patients with prolonged post concussion syndrome (PCS), even years after the brain insult [18–20] (see Discussion section for more details). As explained in the discussion it is plausible that increasing oxygen concentration by HBOT can change the brain metabolism and glial function to rectify the FMS-associated brain abnormal activity. It has already been demonstrated that exposure to hyperbaric oxygen induces significant anti-inflammatory effect in different conditions and pathologies [21–24]. As such, it was also demonstrated that repetitive HBOT may attenuate pain by reducing production of glial cells inflammatory mediators [25, 26].

About a decade ago, Yildiz et al. (2004) [27] found a significant reduction in the number and threshold of tender points following HBOT. The effect of HBOT was not restricted to FMS. Similar improvements following HBOT were reported in complex regional pain syndrome [28–30], idiopathic trigeminal neuralgia [31], migraines [32], cluster headaches [33], and other pain conditions[34, 35]. Studies with animal models also demonstrated that HBOT can relieve pain in chronic pain condition [36, 37].

### The crossover approach

There is a persisting dilemma regarding the adequate sham control for testing the effects of HBOT. The standard requirement for proper sham control is: *“Medically ineffectual treatment for medical conditions intended to deceive the recipient from knowing which treatment is given*.*”* Naively, one could assume that placing the patients in the HBO_2_ chamber at normal air (21% oxygen) and normal pressure (1.0Atm) can serve as proper control. However, in order to have them sense elevated pressure, as required by sham control, the chamber pressure must be increased to 1.3 Atm or higher. The problem is that breathing normal air at 1.3 Atm can elevate the dissolved tissue oxygen by 50% or more, leading to significant physiological effects. Hence, room air at 1.3 Atm is not *“ineffectual treatment”* and cannot serve as proper sham control as required by the placebo definition. We decided to adopt the crossover approach which we had successfully used to test the effect of HBOT in post-stroke patients and in victims of mTBI at late chronic stage [18, 19]. In this approach, the participants are randomly divided into two groups. One, the trial group, receives two months of daily HBO_2_ treatments while the other, the control group, goes without treatments during that time. The latter is then given the same treatments two months later. The study endpoints include, in addition to the physiological evaluations, also blinded detailed computerized clinical evaluations with SPECT scans that were blindly compared for all patients. The advantage of the crossover approach [18, 19] is three-fold, as it allows comparison between treatments of two groups, between treatment and no treatment of the same group, and between treatment and no treatment in different groups. This is further reflected upon in the discussion section.

**The goal** of our current study was to provide firm evaluation of the HBOT effect on brain activity and well-being of FMS patients and to look for correspondence between changes in brain activity as assessed by SPECT imaging and improvements in the FMS symptoms.

## Methods

The protocol for this trial and supporting CONSORT checklist are available as supporting information; see Clinical study S1 Protocol, S1 Consent Form, S1 CONSORT Checklist. The study was performed as a prospective clinical trial conducted at the hyperbaric institute and the research unit of Assaf-Harofeh Medical Center, Israel. Enrolment of patients started in May 2010 and ended in December 2012. The protocol was approved by Assaf-Harofeh institutional review board. All patients signed written informed consent.

### Participants

#### Inclusion

The sixty participants were patients between the ages of 21–67, diagnosed with fibromyalgia at least 2 years prior to the inclusion. The fibromyalgia diagnosis was based on two criteria: (1) Symptoms of widespread pain occurring both above and below the waist and affecting both the right and left sides of the body; (2) Physical finding of at least 11 of 18 tender points.

#### Exclusions

Exclusions were due to chest pathology incompatible with HBOT, inner ear disease, claustrophobia and inability to sign informed consent. Smoking was not allowed during the study.

### Protocol and End Points

After signing an informed consent form, the patients were invited for baseline evaluation. Included patients were randomly assigned to two groups (1:1 randomization): a ***treated group*** and a ***crossover group***. Study endpoints included assessments of tender point count, pain threshold, functional impairment (Fibromyalgia Impact Questionnaire—FIQ)[38], symptom severity (SCL-90 questionnaire)[39] and Quality of life (SF-36 questionnaire)[40, 41]. In addition, the study endpoints included assessment of brain activity according to SPECT imaging. Evaluations were made by medical and neuropsychological practitioners who were blinded to patients' inclusion in the control-crossover or in the treated groups.

**Fig 1:**
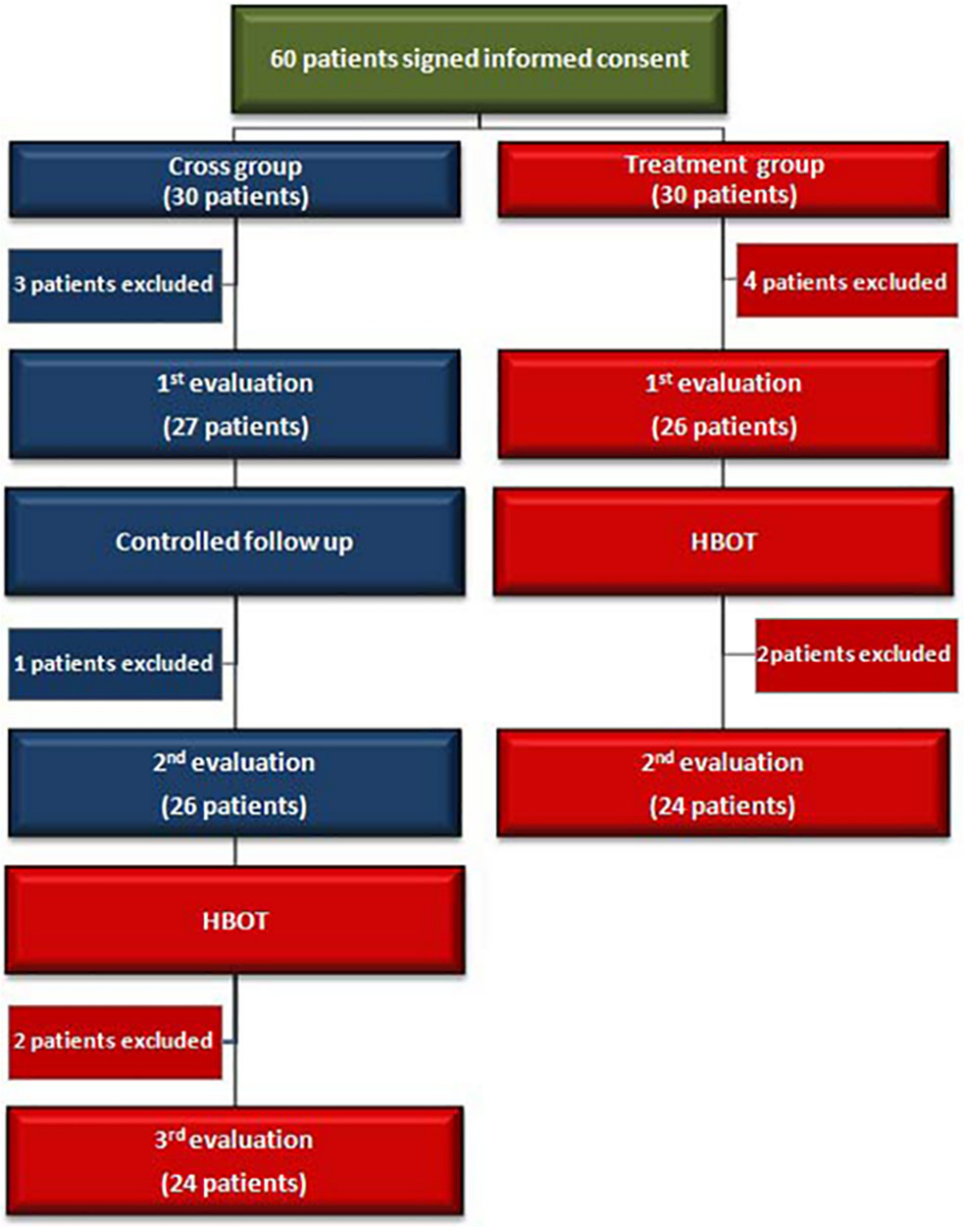
Flow chart of the patients in the study.

Patients in the treated group were evaluated twice – at baseline and after 2 months of HBOT. Patients in the crossover group were evaluated three times: baseline, after 2 months control period (no treatment), and after subsequent 2 months of HBOT (Fig 1). The post-HBOT evaluations as well as the SPECT scans were done more than 1 week (1–4 weeks) after the end of the HBOT protocol. The following HBOT protocol was practiced: 40 daily sessions, 5 days/week, 90 minutes each, 100% oxygen with air breaks at 2.0ATA.

Patients were not involved in any other rehabilitation or pain intervention as part of the study protocol. The detailed clinical study protocol, copy of the informed consent, and the CONSORT 2010 checklist of information are attached as supporting information 1, 2 and 3 (S1 Protocol, S1 Consent Form, S1 CONSORT Checklist). We note that information regarding sample size, detectable change and power calculation parameters is included and addressed in the "statistical considerations" section in the S1.

### Evaluation of the syndrome state

#### Tender point count and pain threshold evaluations

Pain response level was quantitatively evaluated in terms of tender point assessment by a rheumatologist, who was blinded to group assignment. Tenderness was assessed manually and quantified using a dolorimeter. A count of 18 tender points at nine symmetrical sites was performed by thumb palpation. The amount of manual pressure applied to a tender point was about 4 kg/cm^2^ (tested with a dolorimeter). Thirteen point sites (nine tender point sites and four control sites) were further studied using a dolorimeter. The threshold of tenderness was measured with a Chatillon dolorimeter, model 719–20, which has a maximum scale of 9 kg, with a neoprene stopper footplate with a diameter of 1.4 cm (Pain Diagnostics & Thermography Inc.,New York, USA)[41]. All dolorimeter measurements of the 13 point sites, as well as a total point count, were done by one rheumatologist (D.B), who was blinded to patient group.

#### Functional impairment

A validated Hebrew version of the Fibromyalgia Impact Questionnaire (FIQ)^38^ was used to evaluate the level of functional impairment. The first part of the FIQ focuses on the patient’s ability to perform daily tasks (i.e. driving, cleaning, etc.) and contains 10 items with responses ranked 0 to 3, where 0 = “always able”, and 3 = “never able”. The item scores were normalized to range from 0 to 10 for uniformity, with 10 representing worst physical function. The mean of the items yields a single physical function score. Internal consistency of the FIQ questionnaire was computed using internal consistency Cronbach alpha measure. The reliability was α = 0.844 on the first time-point of data collection, and α = 0.907 on the second time point of data collection.

#### Psychological distress

The Symptom Check List (SCL-90)[39] was used to examine the level of psychological distress. This questionnaire consists of 90 items measuring 9 clinical subscales: somatisation, obsession-compulsion, interpersonal sensitivity, depression and anxiety, hostility, phobic anxiety, paranoid ideation and psychoticism. Each subscale is assigned a 5-point Likert scale from 0 to 4 with a higher score corresponding to higher distress. Internal consistency of the SCL-90 questionnaire was computed using internal consistency Cronbach alpha measure. We found a very high reliability with α>0.95.

### Quality of life evaluation

Quality of life (QoL) was evaluated by the questionnaire SF-36 [40, 41]. This health-related QoL measure contains 36 items, and health status is assessed across three domains: functional status, well-being and overall evaluation of health. The Hebrew translation of the SF-36 was validated in an adult general population, and our group has further evaluated the Hebrew version on patients with widespread pain, both associated and not-associated with FMS[42]. The SF-36 contains eight subscales: physical functioning, social functioning, and role limitations attributable to physical and emotional problems, mental health, vitality, bodily pain and general health. Each scale generates a score from 0 to 100, with a high score indicating better health and less body pain. Internal consistency of the SF-36 questionnaire was computed using internal consistency Cronbach alpha measure. We found a very high reliability with α>0.85.

### Brain Functional Imaging

Brain single photon emission computed tomography (SPECT) was conducted with 925–1,110 MBq (25–30 mCi) of technetium-99m-methyl-cysteinate-dimmer (Tc-99m-ECD) at 40–60 min post injection using a dual detector gamma camera (ECAM or Symbia T, Siemens Medical Systems) equipped with high resolution collimators. Data was acquired in 3-degree steps and reconstructed iteratively with Chang method (μ = 0.12/cm) attenuation correction [43].

Regional cerebral blood flow change analysis was conducted by fusing pre- and post-treatment studies that were normalized to median brain activity. SPECT images were reoriented into Talairach space using NeuroGam (Segami Corporation) for identification of Brodmann cortical areas and in order to compute the mean perfusion in each Brodmann area (BA). All SPECT analyses were done while blinded to the laboratory and clinical data.

#### Changes, average changes and normalized average changes

Changes in perfusion in all Brodmann areas for each subject were determined by calculating the percentage difference between post-period and pre/baseline-period divided by the pre/baseline-period perfusion. The relative change, R_change_(i,n) of Brodmann area (n) for patient (i), is defined as:

$$Rchange(i,n)=\frac{\left[PostA(i,n)-PreA(i,n)\right]}{\left[PreA(i,n)\right]}$$

Where PostA(i,n) and PreA(i,n) represent the normalized activity of the n^th^ Brodmann area at the end point and start point of the assessment period (either treatment or control) respectively. Note that when multiplied by 100, R_change_(i,n) is the percent difference.

An averaged relative change,

<Rchange>(n)=<Rchange(i,n)>i

was calculated for each Brodmann area for each group according to study phase (control and treatment periods of the crossover group and treatment period of the treated group).

#### Response group

To inspect the association between changes in the brain activity according to SPECT imaging and changes in the syndrome severity, we divided the 48 patients into two subgroups according to their response to the treatment. More specifically, we use the changes in the number of tender points and the level of threshold pressure as classifiers. The 41 patients which exhibited improvements in these parameters were classified as responders (physiologically improved), and were assigned to a response group. The other 7 patients were classified as non responders and were assigned to a non response group.

### Significance index

Brain activity is signified by variations between the different brain’s locations, and these variations change over time according to the tasks performed. These inherent spatiotemporal variations are reflected by high variance in the brain activity at each Brodmann area, as measured by SPECT imaging. The statistical challenge imposed by the SPECT imaging is the low signal-to-noise ratio: that the magnitude of the non arbitrary changes in the brain activity (following treatment) in most of the Brodmann area are comparable to the magnitude of the arbitrary change related to the inherent person-to-person and time variations that are not related to the treatment.

To meet the challenge, we introduced a significance index I_σ_(n) to substantiate the comparison between the changes in brain activity in the response group during treatment and those in the crossover group during the control period. We defined I_σ_ (n)as:

Iσ(n)={PC(n)*[1-PR(n)]}12

where P_C_(n) is the p-value of the change in SPECT measurements (calculated in two-tailed t-test) for the post control vs. pre control period of the patients in the crossover group. Similarly, P_R_(n) is the p-value of the change in SPECT measurements for the post treatment vs. pre treatment period of the patients in the response group (the responders). The rationale for the new index is that lower values of P_R_(n), hence higher values of [1-P_R_(n)], correspond to higher significance of the changes during treatment. On the other hand, higher values of P_C_(n) imply that the changes during control are likely to vary arbitrarily prior to treatment. Hence, consistent changes measured during treatment are more significant. The significance index is defined such that both contributions are included. We tested other putative definitions of the significance index—for example, {[P_C_(n)]/[P_R_(n)]}

^1/2^ that represents the ratio between the significance of the changes during treatment vs. the changes during control—and obtained similar results.

### Statistical Analysis

SPSS software (version 19, IBM Inc.) was used for the statistical analyses. Continuous data is expressed as means ± standard deviations (STD). For each dependent measure, an analysis of variance was performed according to the time-point of data collection (before vs. after HBOT) and according to the associated group (treated vs. crossover) as independent measures. Additionally, repeated one-way analysis of variance was computed using the three time-points of data collection for the crossover group. When relevant, post hoc comparisons were used as is reported in the results section. Categorical data is expressed in numbers and percentages and compared by chi-square test. With regards to dolorimeter thresholds analysis, an average of thresholds was calculated for each patient, and this average was used in the ANOVA model.

Sample size was based on the assumption that exposure to the Dolorimeter evaluation (at baseline) without any additional training might induce up to 8% (0.06 Dolorimeter change) improvement in the second Dolorimeter evaluation (following HBOT), based on Yildiz et al. [27]). A threshold of tender sites was selected as a criterion for sample size since this was the smaller anticipant effect. The sample size was calculated to provide 80% power to show that HBOT induces at least 87% improvement on Dolorimeter threshold of the tender sites. This was based on a power analysis using the normal approximation for the binomial, with one-sided Alpha = 0.05. Note that it is based on a cross over design without sequence effect.

### Registration

The study was officially registered in ClinicalTrials.gov, Identifier: NCT01827683, after patients enrolment started due to technical delay. The authors confirm that all ongoing and related trials for HBOT in fibromyalgia are registered.

## Results

The study was conducted between May 2010 and December 2012. Sixty female patients signed a written informed consent. Eight patients were excluded before the hyperbaric oxygen treatment and additional four patients were excluded during treatment.

### Pre-study exclusions

Seven patients refused to enter the hyperbaric chamber before the beginning of the control/treatment period (3 in the crossover group and 4 in the treated group). One patient was excluded in the crossover group during the control period.

#### In-study exclusions

Four patients decided to drop out during the treatment protocol due to dizziness, claustrophobia and inability to adjust by “ear pumping” to the hyperbaric condition (2 in the crossover group and 2 in the treated group).

Accordingly, 48 patients (24 in the treated group and 24 in crossover group) were included in the final analysis (Fig 1). All patients were females of ages 21–67, and the time elapsed from the FMS diagnosis to the study recruitment was 2–22 years with mean of 6.5 years.

#### Baseline characteristics

Patients’ characteristics are summarized in Table 1. As seen from this table, there was no significant difference in the included measures between the two groups.

**Table 1:** Demographic of patients' characteristics.

	Treated Group	Crossover Group	p Value
	(n = 24)	(n = 26)	
**Age** (years)	50.4±10.9	48.1±11.1	0.677
**Years of education**	17.1±3.5	14.8.±3.0	0.019
**Duration of fibromyalgia** (years)	6.75±5.9	6.2±5.1	0.735
**Number of children**	2.38±1.21	2.95±1.43	0.156
**Marital status:** Married	21 (87.5%)	18 (69.2%)	0.239
Single	1 (4.1%)	5 (19.2%)	
Divorce	2 (8.3%)	1 (3.8%)	
Widow	0 (0%)	1 (3.8%)	
**Life style:** Secular	19 (79.2%)	17 (65.3%)	0.662
Traditional	4 (16.6%)	6 (23.1%)	
Religious	1 (4.1%)	2 (7.6%)	
**Place of born:** Israel	20 (83.3%)	18 (69.2%)	0.297
USSR	0 (0%)	2 (7.6%)	
else	4 (8.3%)	6 (23%)	
**Economic status:** Very bad	0 (0%)	1 (3.8%)	0.77
Bad	2 (8.3%)	2 (7.6%)	
Medium	16 (66.7%)	18 (69.2%)	
Very good	6 (25%)	5 (19.2%)	
**Work**	16 (66.7%)	17 (77.3%)	0.425
**Body Mass Index** (kg/m2)	26.9±5.8	27.2±4.7	0.849
**Diabetes Mellitus**	1 (4.1%)	2 (7.6%)	0.55
**Dyslipidemia**	9 (37.5%)	10 (38.5%)	0.859
**Hypertension**	6 (25%)	5 (19.2%)	0.671

### The Effect on Pain

#### Tender point evaluation

**Fig 2:**
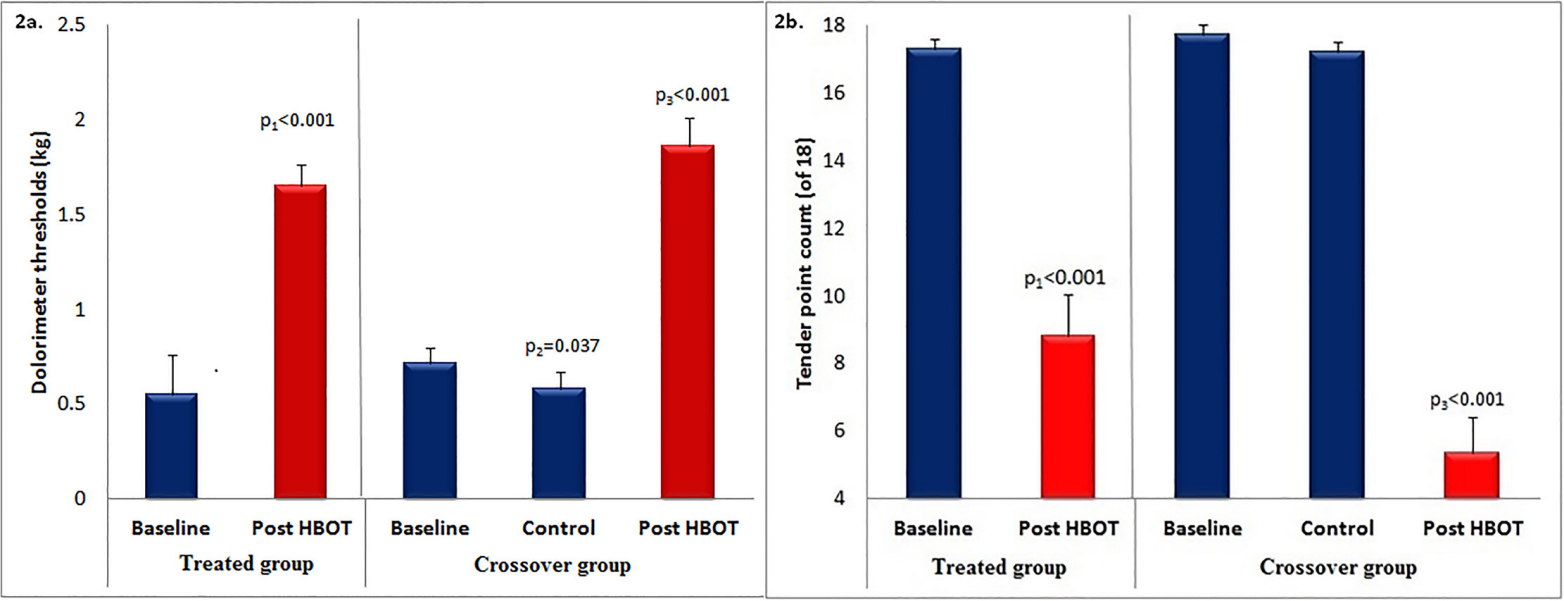
The HBOT effects on tender points. A) The effect on dolorimeter threshold. For both groups, the threshold level tripled after treatment (about 1.5, red bars, vs. about 0.5, blue bars). B) The effect on the number of tender points. The treatment led to significant reduction in the number of tender points in both groups: by a factor of 2 in the treated group and by a factor of 3 in the crossover group.

The effect of the hyperbaric oxygen treatment on the patients’ pain, as assessed by the change in the dolorimeter threshold of the tender points (see Methods) is summarized in Fig 2 and in Table 2. Fig 2A shows the treatment effect on the dolorimeter thresholds and Fig 2B shows the effects on the number of tender points. It is transparent in the figure that the two groups had very close mean scores at baseline for both measures (within the standard error). It is also transparent that the HBOT treatments of both groups led to statistically significant improvements in the mean scores of both the dolorimeter thresholds and of the number of tender points.

**Table 2:** Summary of the results of the tender points evaluation, physical function assessment, symptoms and quality of life questionnaires.

	Treated group			Crossover group					Between Groups
	Baseline	Post HBOT	P_1_	Baseline	Control period	Post HBOT	P_2_	P_3_	P_4_
**Tender point count (of 18)**	17.33±1.4	8.87±6.03	<0.001	17.71±0.69	17.24±1.15	5.35±4.47	0.56	<0.001	<0.001
**dolorimeter thresholds(kg)**	0.55±1.7	1.65±0.81	<0.001	0.72±0.46	0.58±0.46	1.86±0.76	0.037	<0.001	<0.001
**(9 tender sites)**									
**Dolorimeter thresholds(kg)**	2±0.75	3.24±1.05	<0.001	2.19±0.51	1±0.53	2.29±0.76	0.05	<0.001	<0.001
**(4 control sites)**									
**Physical Function Assessment (FIQ score)**	3.76±0.73	2.51±1.14	<0.001	3.76±1.06	3.7±1.15	2.71±1.12	0.876	0.02	0.001
**Symptom Check List**	0.88±0.47	0.66±0.4	0.004	1.23±0.64	1.08±0.62	0.71±0.27	0.296	0.009	0.009
**(SCL-90 score)**									
**Quality of life**	3.15±0.44	3.48±0.45	<0.001	2.89±0.47	3.03±0.38	3.32±0.36	0.1	0.01	<0.001
**(SF-36 score)**									

P_1_- p values for comparison before and after HBOT in the treated group (paired t test).

P_2_- p values for comparison before and after the control period in the crossover group (paired t test).

P_3_- p values for comparison after the control period before and after HBOT in the crossover group (paired t test).

P_4_- p values for comparison of the treated group after HBOT and the crossover after the control period (independent sample t test).

* Data is presented as mean± standard deviation

As seen in Fig 2 and detailed in Table 2, the dolorimeter threshold score significantly improved following HBOT in the treated group (mean change 1.11±0.79, p < 0.001) and in the crossover group after HBOT (mean change 1.29±0.76, p < 0.001). Effect sizes were large: the Cohen’s d measures were 1.3 and 1.68, respectively. The number of tender points was significantly reduced following HBOT in the treated group (mean change 8.46±5.36, p < 0.001) and in the crossover group after HBOT (mean change 11.54±4.93, p < 0.001). The effect sizes were large: Cohen's D measures were 1.5 and 2.24, respectively.

As expected, no improvement was noticed in the crossover group following the control period, neither in the dolorimeter thresholds nor in the point count. It can be seen that the crossover group had the same general score at baseline and after the control period. This value seems higher than the score of the treated group at baseline – 0.65 vs. 0.55, and the post-HBOT dolorimeter thresholds score of the treated group seems lower than that of the crossover group – 1.65 vs. 1.85

#### Examining the relative changes

**Fig 3:**
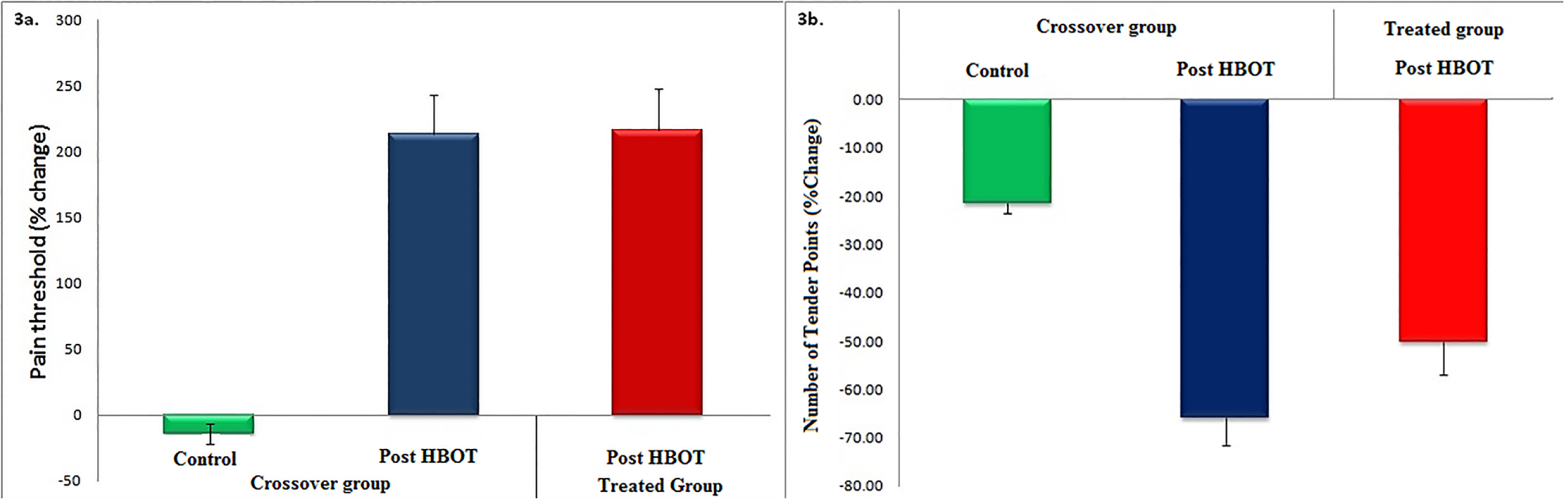
Assessments of the mean relative changes in the pain level. A) The mean relative change and standard errors in the dolorimeter thresholds for the crossover group following the control period (green) and following HBOT (blue), and for the treated group following HBOT (red). B) The mean relative changes and standard errors in the number of tender points for the crossover group following the control period (green) and following HBOT (blue), and for the treated group following HBOT (red).

There is a high patient-to-patient variability in the dolorimeter thresholds. The magnitude of the change in a dolorimeter threshold has different implications for patients at low or high base levels. Hence, we inspected the effect of the HBOT on the relative change, i.e., the change relative to the base value. We calculated, for each person, the relative change in the dolorimeter threshold for each period (control and HBOT for the crossover group and HBOT for the treated group). In Fig 3A we show the mean relative changes in dolorimeter threshold for the crossover group following the control period and following HBOT, and for the treated group following HBOT. We note that calculating the mean of the relative changes is more informative than calculating the changes in the mean values, especially for small groups with high patient-to-patient variability. Looking at the relative changes elucidates the improvements after the HBOT period vs. the control period of the crossover group and the baseline for the treated group. The same analysis was conducted for the number of tender points. In Fig 3B we show the mean relative changes in the number of tender points for the crossover group following the control period and following HBOT, and for the treated group following HBOT. For the control group, we also compared between the relative changes during the control + treatment periods (the combined period) and during the treatment period and found them statistically equal (S1 File).

#### Scatter plot analysis of the dolorimeter threshold

**Fig 4:**
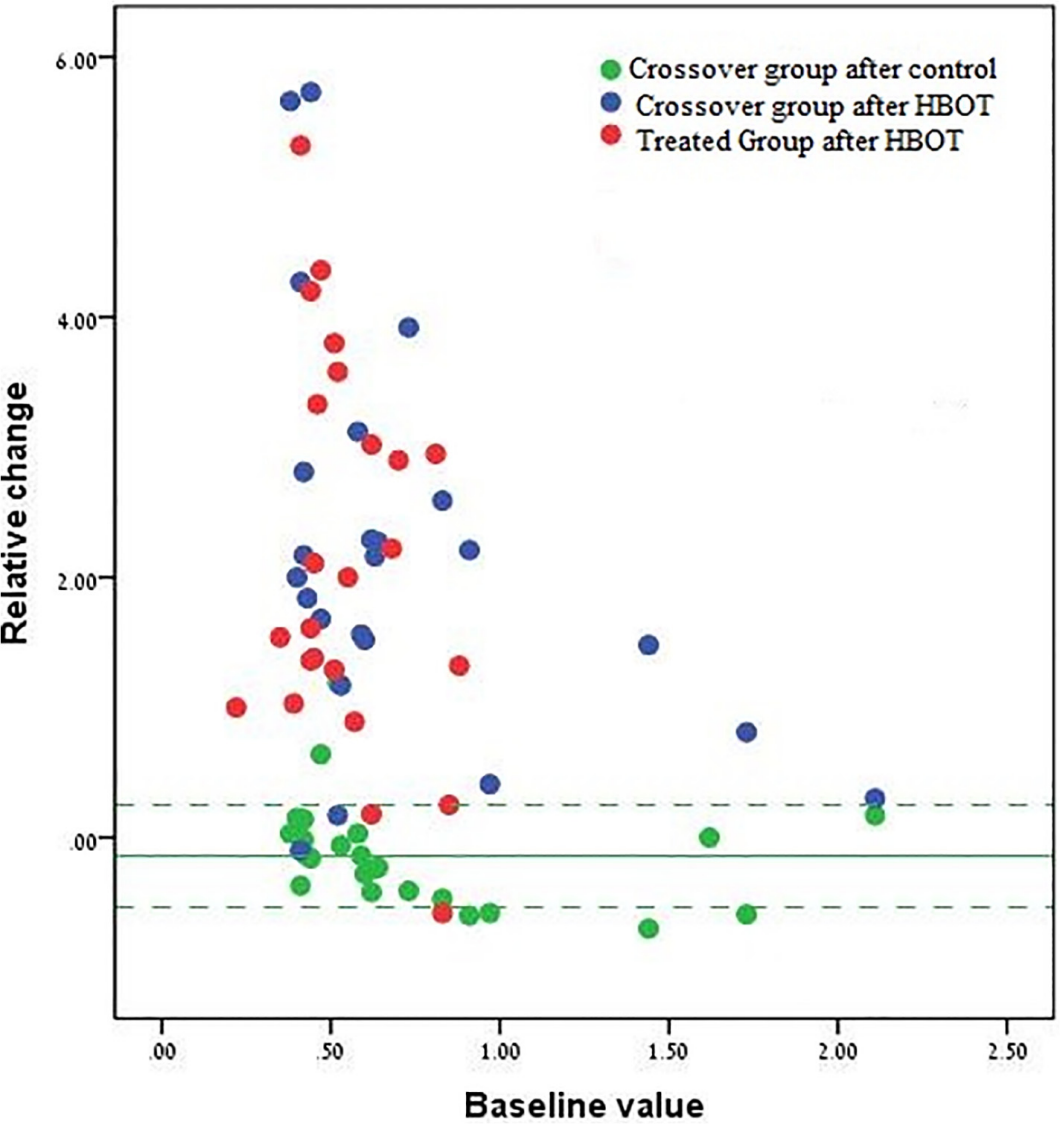
Scatter plot of the individual relative changes in the dolorimeter threshold. The figure shows the relative change in all patients (y-axis in unit change) as a function of the baseline value. For the treated group, each patient is represented by a single red dot. The relative change is the change during HBOT and the baseline value is the value before treatment. For the crossover group, each patient is represented by two dots: a green dot represents the relative change during the control period, with the baseline being the value before the control. A blue dot represents the relative change during treatment, with the baseline value being the value before treatment (which is also the value at the end of the control period). The green line represents the mean relative change in the crossover group following the control period and the green dashed lines represent the ±1std from the mean.

In Fig 4, we show a scatter plot of the relative changes in dolorimeter threshold as a function of baseline. The results illustrate the differences between the control period of the crossover group and the post HBOT of both groups. Notably, apart from 6 patients (3 from the crossover group and 3 from the treated group), all others showed significant improvement following the treatment. Note that, in general, the higher the baseline threshold the smaller the improvement.

### The Effects on Physical Functions, Psychological Distress and Quality of Life

The HBOT effects on the physical functions, the psychological distress and the quality of life are detailed in Table 2.

#### Physical function assessments

The FIQ score significantly improved following HBOT in the treated group (mean change 1.31±0.99, p < 0.001) and in the crossover group after HBOT (mean change 1.02±0.92, p = 0.05). The effect sizes were large and medium: Cohen's D measures were 1.29 and 0.64, respectively. As expected, there was no improvement in the FIQ score in the crossover group following the control period.

#### Psychological distress

The SCL-90 score significantly improved following HBOT in the treated group (mean change 1.10±0.79, p < 0.01) and in the crossover group after HBOT (mean change 1.29±0.76, p = 0.05). The effect sizes were medium: Cohen's D measures were 0.66 and 0.60, respectively. As expected, there was no improvement in the SCL-90 score in the crossover group following the control period.

#### Quality of life assessments

The SF-36 score significantly improved following HBOT in the treated group (mean change 0.34±0.33, p < 0.01) and in the crossover group after HBOT (mean change 0.23±0.39, p = 0.05). The effect sizes were large medium: Cohen's D measures were 1.0 and 0.58, respectively. As expected, there was no improvement in the SF-36 score in the crossover group following the control period.

#### Examining the relative changes

**Fig 5:**
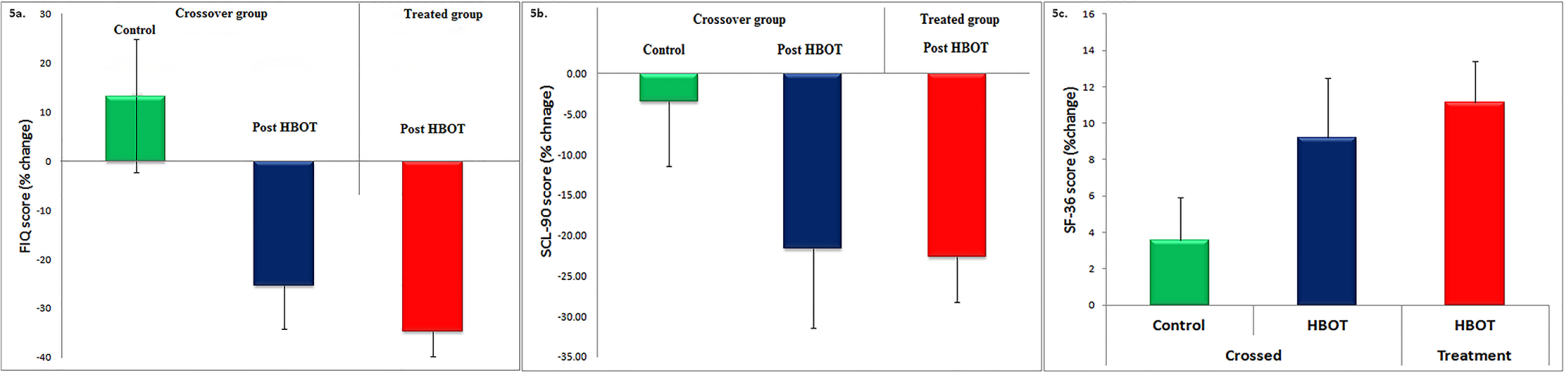
Assessments of the mean relative changes in the FIQ, SCL-90 and the SF-36 scores. The figures show the mean relative changes and standard errors in the three measures for the crossover group following the control period (green) and following HBOT (blue), and for the treated group following HBOT (red). A) Mean relative changes and standard errors in physical function assessed by the FIQ score. B) Mean relative changes in and standard errors in the psychological distress assessed by the SCL-90 score. c) Mean relative changes and standard errors in the quality of life assessed by the SF-36 score.

Similar to the pain related scores, there is also a high patient-to-patient variability in the FIQ, SCL-90 and the SF-36 scores. Hence, we also inspected the effect of the HBOT on the relative changes in these scores. The results shown in Fig 5 reveal significant improvements in all scores following treatment for both groups. In S1 File we show a comparison between the relative changes in FIQ, SCL-90 and SF-36, during the combined and the treatment periods for the patients in the crossover group (see definition in the effect on pain section above).

### SPECT assessments of changes in brain activity

#### Motivation

As mentioned in the introduction, earlier studies compared SPECT images of FMS patients to those of healthy subjects. The studies revealed a notable difference in brain activity between the two groups. In particular, they found that FMS is associated with elevated activity in the somatosensory cortex and reduced activity in the frontal, cingulate, medial temporal and cerebellar cortices [11, 12]. These results provide an excellent independent control reference to which changes in brain activity following HBOT should be compared to.

#### Within group and between groups comparison

The crossover affords two types of comparison: 1. within group—between the changes in FMS symptoms and in brain activity during the control period and during the treatment period in the same patients (of the crossover group). 2. between groups – between the changes during treatment in patients of the crossover group vs. patients of the treated group. Even more persuasive was the correspondence we found between the brain areas whose activity increased/decreased following the HBOT sessions and the brain areas that were shown in previous studies to have reduced/enhanced activity in FMS patients relative to normal subjects. In order to attain greater validity, symptom assessment and SPECT analysis were done by blinded evaluations and evaluators: the tests of the FMS state were done by computerized validated methods and the SPECT analysis was blind to patients' participation in treated/crossover group.

#### Association

Brain SPECT imaging was performed and evaluated for all patients. The patients in the treated group had two SPECT imagings (pre- and post-treatment) and the patients in the control group had three SPECT imagings (pre- and post-control period, and post-treatment). One patient from the control group missed the post-control SPECT imaging (hence we have 23 results for SPECT assessed brain activity during the control period). In S2 File we present detailed results of SPECT imaging for all Brodmann areas (BAs) of all the tested patients. NeuroGam software, used to normalize and average the SPECT measurements into Brodmann areas, excludes small volume BAs from the available data in order to avoid inconsistent results. Therefore, the following BAs were not assessed in this study: Bilateral 1, 2, 3, 12, 26, 29, 30, 33, 34, 35, 41, 42, 43, 48, 52.

#### Association vs. correlation

We specifically use the term “association” rather than “correlation” since direct mathematical correlations between the physiological changes and the changes in brain activity are ill defined—there is no one-to-one correspondence between the Brodmann areas and the physiological functions, as each physiological function can be performed by locations spread over several Brodmann areas and vice versa. We would like to emphasize that even in the cases that correlation can be defined and computed, correlations do not reveal causality. Moreover, from biological perspective, the changes in the brain activity are expected to cause physiological changes that in turn can lead to additional changes in the brain activity. Therefore, our aim was to show correspondence, rather than mathematical correlations, between the changes in the brain activity and the physiological changes.

#### BA histogram of mean relative changes

**Fig 6:**
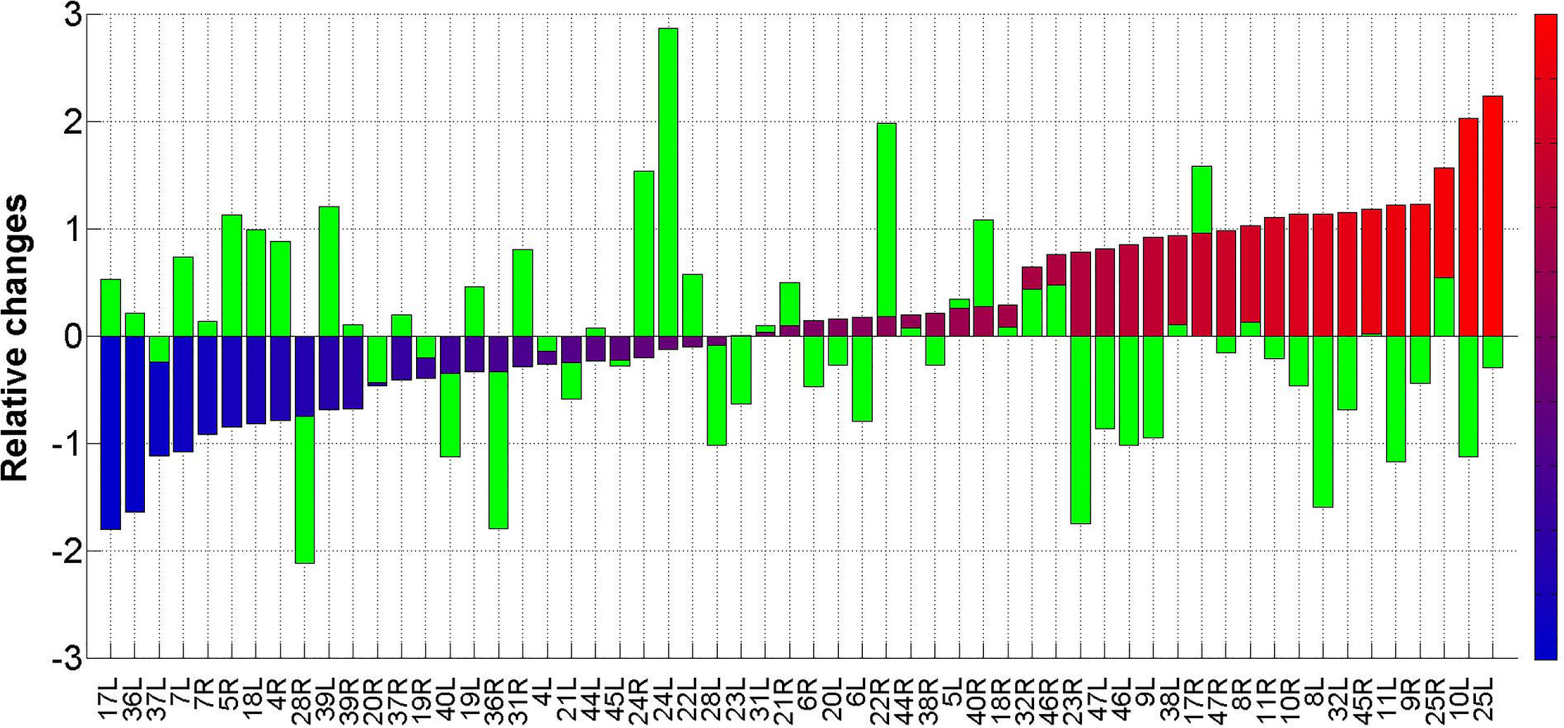
BA histogram of mean relative changes. The figure shows the histogram as is explained in the text. The Y-axis shows the mean relative change <R_change_>(n) for the Brodmann area indicated in the X-axis. The results for the patients of the response group after the HBOT period are colored from light blue (BA with the strongest activity reduction) to light red (BA with the highest activity elevation). The green bars correspond to the mean relative changes in the patients of the crossover group following the control period.

To summarize and assess the results, we constructed histograms of the mean relative changes, <R_change_>(n), for each Brodmann area (n). To construct the results shown in Fig 6, we calculated, for each patient (i), the relative change in the SPECT measured brain activity, R_change_(i,n), during each phase of the trial (see Methods section). Then we calculated the average changes, <R_change_>(n), for the 41 patients (out of 48) from the treated group and the crossover group that showed significant improvement in the FMS symptoms following HBOT (the response group mentioned in the method section) and ordered the results from the most reduced to the most elevated activity. The changes in the BAs of te response group following HBOT were compared with those of the patients in the crossover group during the control period.

To quantify the results shown in Fig 6 and illustrate the statistical significance, we also calculated the Pearson correlations for the following four combinations. 1. The correlations between the vectors of the mean relative changes for the response group and the vectors for the crossover group during the control period. 2. The correlations between the mean relative changes during treatment for the group of 41 responders and those for the group of 7 non responders. 3. The correlations between the mean relative changes during treatment for the whole response group and those for the responders from the treated group. 4. The correlations between the mean relative changes during treatment for the whole response group and those for the responders from the crossover group. The correlations for the four combinations were found to be -0.25, -0.05, 0.77 and 0.68, respectively.

#### Normalized BA histogram of mean relative changes

**Fig 7:**
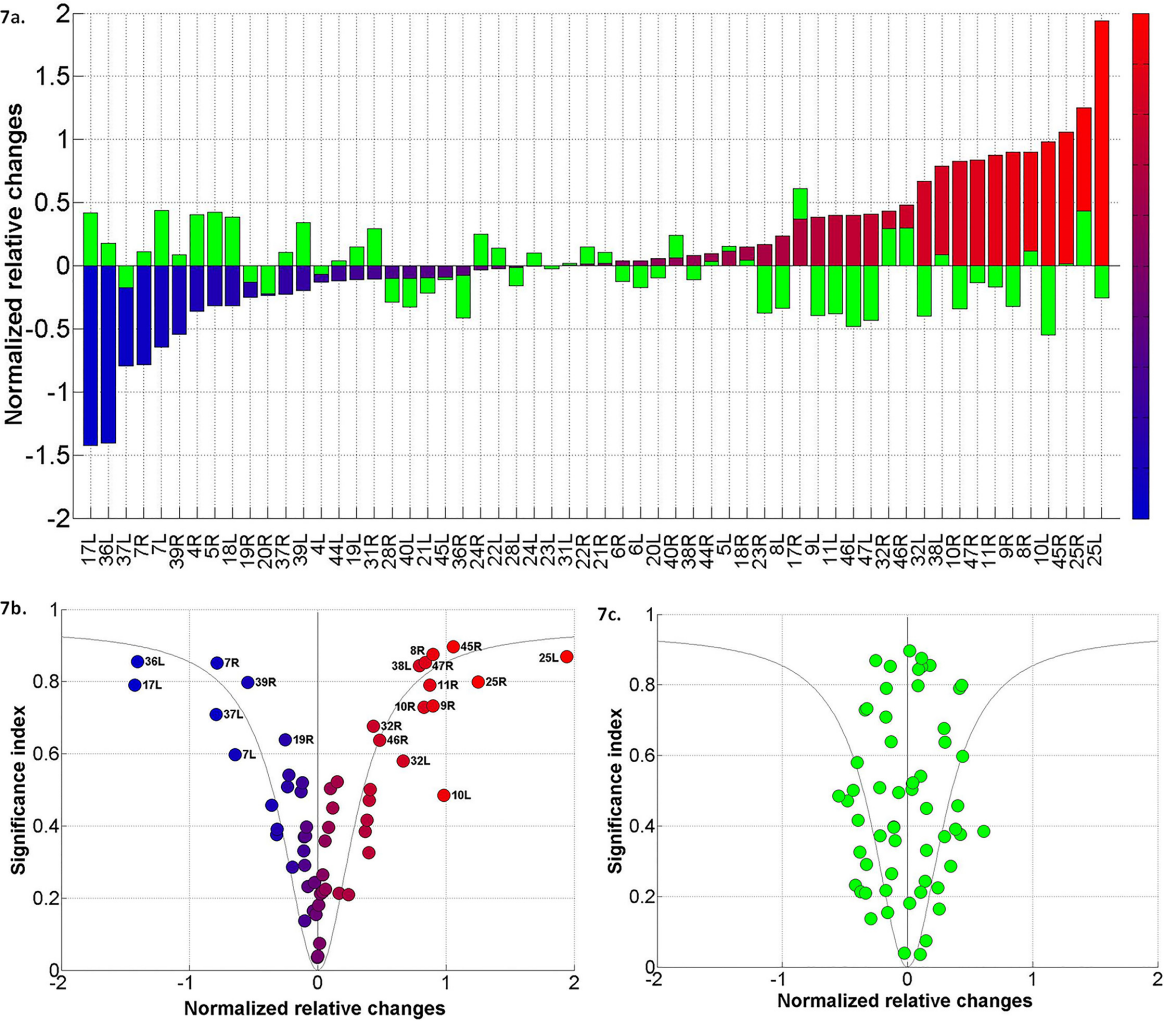
The effect of significance index normalization. A) Normalized BA histogram of mean relative changes. The figure is similar to Fig 6 but the Y-axis is for the normalized values, that is for I_σ_(n)* <R_change_>(n) and not for <R_change_>(n) that are used in Fig 6. The BAs within the rectangles are the ones with normalized mean relative changes smaller than -0.6 or larger than +0.6. B) The two dimensional scatter plot I_σ_(n) vs. I_σ_(n)* <R_change_>(n) for the patients of the response group following the HBOT period. C) Similar scatter plot for the patients in the crossover group following the control period. The color code in (B) and (C) is the same as in (A). The funnel shaped black curve is a fit of the results in (B) to a reciprocal Lorentzian curve: f(x) = {X_max-_ γ*[π*(γ^2^+x^2^)]^-1^} with X_max_ = 0.95, γ = 0.335.

In Fig 7A we show a histogram similar to the aforementioned one, but in which we normalized the mean relative changes of each BA (n) by its corresponding significance index I_σ_(n) as is defined and explained in the Methods section. To better scrutinize the effect of the normalization, we constructed 2-dimentional scatter plots of the significance index vs. the normalized relative changes. In Fig 7B we show the results for the patients in the response group following the HBOT period; in Fig 7C we show the results for the patients in the crossover group following the control period. Comparison between the two scatter plots reveals that, following treatment, the Brodmann areas that show large changes in brain activity also have high significance factors (see Figs 6 and 7B). In contrast, comparing Fig 7C and 7A reveals that, following the control period, the significance index is low for Brodmann areas that exhibit big changes in brain activity. The correlations for the four combinations mentioned above, calculated for the normalized mean changes, were found to be -0.28, -0.09, 0.66 and 0.61, respectively.

### Assessment of the results

**Fig 8:**
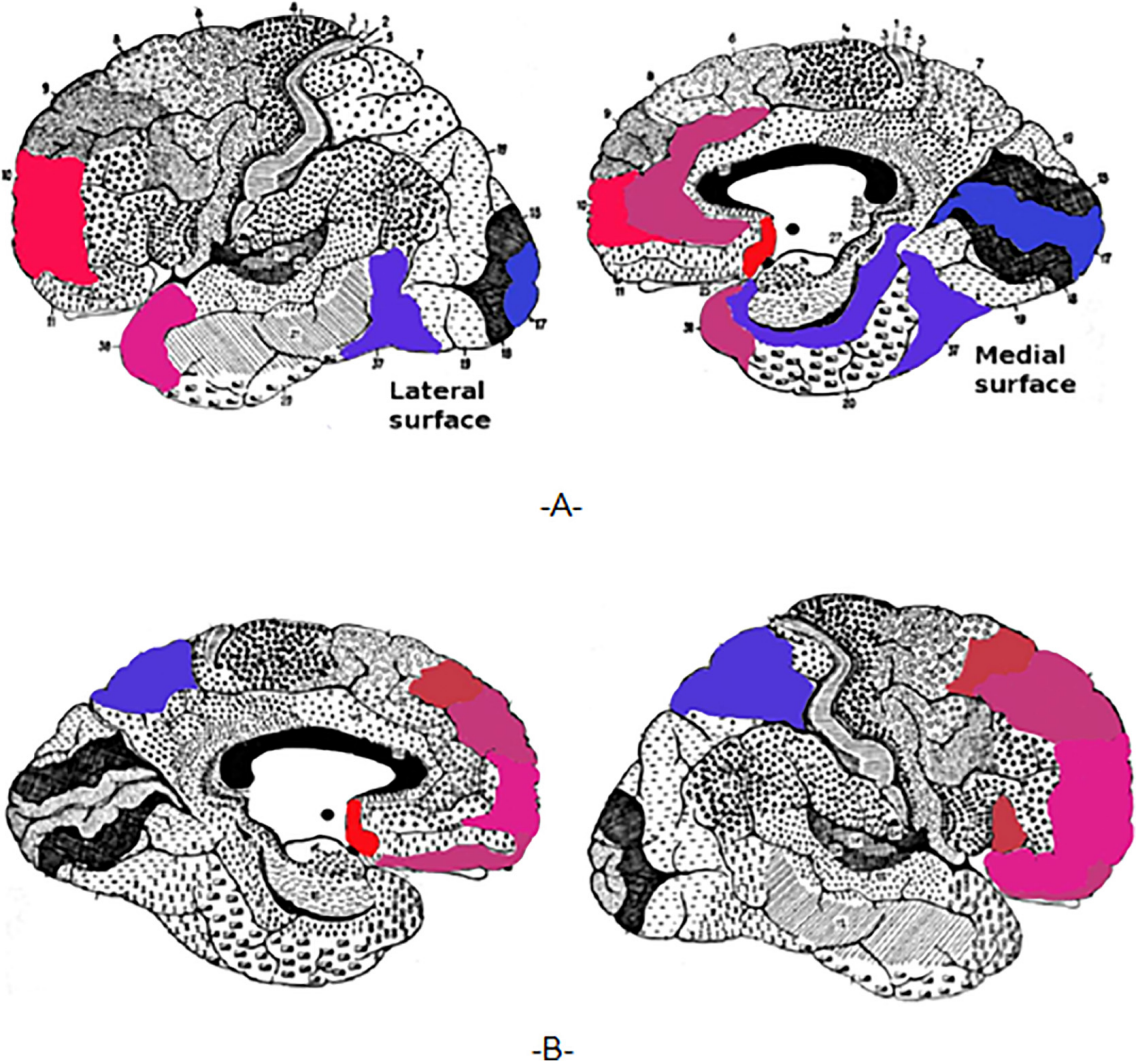
Projection of the significant changes on the brain maps. The figure shows the results of the normalized mean changes as projected on the brain maps, left BAs (A) and right BAs (B). We colored the BAs that show significant changes in activity using the same color code as in Figs 6 and 7 - from light blue (BA with the strongest activity reduction) to light red (BA with the highest activity elevation).

The results in Fig 7 reveal several distinct Brodmann areas with significant normalized changes in the brain activity following the HBOT period. More specifically, in the response group, 10 BAs showed above +0.6 normalized mean changes (hyper-perfusion) and 5 BAs showed below -0.6 normalized mean changes (hypo-perfusion) following the HBOT period. In contrast, the normalized mean changes in brain activity for all BAs are scattered within the (-0.6 — +0.6) range following the control period of the patients in the crossover group. In addition, the scatter of the normalized mean changes after HBOT fits a distinct funnel shape distribution (Fig 7B) that is significantly different from the distribution after the control period (Fig 7C). In Fig 8 we show a projection of the aforementioned findings on the brain maps. For clarification, we used the same color code as in Fig 7.

The results revealed that following the HBOT period, improved patients (responders) exhibit elevated activity of BAs in the frontal lobe (25L+R, 10L+R, 47R, 45R, 11R, 9R, 8R) and in BA 38L, and reduced activity of BAs in the posterior brain (7L+R, 37L, 36L, 17L). As mentioned before, earlier studies showed that FMS patients have reduced brain activity in BAs in the frontal cortex and elevated activity in the posterior brain ^11, 12^. We found that, after treatment, BAs in the posterior brain show reduced activity and BAs in the frontal cortex show elevated activity. Hence, our finding indicate that, in FMS patients, hyperbaric oxygen therapy leads to beneficial changes in the brain activity of specific BAs known to have abnormal activity in these patients.

In the next section we mention that the amelioration consequent to HBOT led to a significant decrease in the intake of pain medications by the patients. In principle, part of the observed changes in the SPECT imaging may be associated with the changes in the intake of pain medication. While this possibility cannot be ruled out, we deem it unlikely. First, we note that the patients have been taking pain medication for a long time (years). The intake of the drugs eased the pain but did not reverse the condition, while HBOT did reverse the condition. Also, the changes in the brain activity as detected by the SPECT coincided with improvement of the FMS symptoms, so much so that most of the patients could reduce or stop altogether the intake of pain medications. In other words, the plausible causal chain is that the changes in brain activity were induced by the HBOT, these changes alleviated the FMS symptoms and eased the pain, leading to a diminished need for pain medication.

### Changes in intake of pain related medications

The amelioration of pain consequent to HBOT led to a significant decrease in the level of analgesic medications intake by the patients in both groups. More specifically, 9 patients from the treated group were on chronic daily medication with analgesic drugs (5 were taking two different drugs and 4 were taking one) before HBOT. After the HBOT, 3 patients got completely off medication, 3 out of the 5 continued with two drugs, and 3 out of the 4 continued with one drug, p = 0.02. In the crossover group, 12 patients were on chronic daily medication of analgesic drugs before HBOT (2 on two drugs and 10 on one drug). All of them continued taking the medications during the control period. Consequent to the HBOT period, 5 patients stopped taking drugs altogether and all other 7 patients took one drug, p = 0.02. With regard to chronic use of antidepressants, in the treated group, the 7 patients that were chronically treated before HBOT continued with their medications at the end of the treatment. In the crossover group, of the 12 patients treated with antidepressants at baseline and during the control period, 8 continued with their medications after the HBOT treatments, p = 0.04.

### Safety and side effects

Five patients decided to stop the HBOT due to dizziness, claustrophobia and inability to adjust ear pressure by “ear pumping”. Thirteen patients had mild barotrauma that resolved spontaneously and did not prevent them from completing the treatment protocol.

Noticeably, 14 patients (29%) reported an increase in the pain/sensation during the first 10–20 session. However, at the end of the HBOT period, all of these patients experience significant amelioration of pain and improvements in the different evaluated parameters in this study as compared to baseline.

## Discussion

We presented a prospective active control, clinical trial of evaluating the effect of HBOT on female patients of ages 21–67 with chronic FMS. The time elapsed from FMS diagnosis to study recruitment was 2–22 years (mean 6.5 years). A crossover approach was adopted in order to overcome the HBOT inherent sham control problem (see discussion further below). The participants were randomly divided into two groups. One, the treated group, received two months of HBOT; the other, the control group, was not treated during those two months and received treatment in the following two months. The advantage of the crossover approach is the option for a triple comparison – between treatments in two groups, between treatment and no treatment in the same group, and between treatment and no treatment in different groups.

The changes in all measures (pain threshold, number of tender points, FIQ, SCL-90 and SF-36) were assessed by detailed computerized evaluations and were compared to changes in brain activity obtained by SPECT imaging. The HBOT in both groups led to similar significant improvements. No significant changes were detected during the non-treatment period in the crossover group. These results are in agreement with earlier findings by Yildiz et al. [27]. Analysis of brain imaging showed significantly increased neuronal activity after a two-month period of HBOT, compared to the control period.

### Brain functionality

What makes the results particularly convincing is the good correspondence between the physiological improvements and the changes in brain functionality as detected by the SPECT scans, as well as the good agreement with the abnormal brain activity of FMS patients. As presented in the introduction, comparison between brain activities of healthy subjects and FMS patients, assessed by SPECT imaging, revealed higher activity in the somatosensory cortex and reduced activity in the frontal, cingulate, medial temporal and cerebellar cortices in FMS patients [11, 12]. We also mentioned that these results are in agreement with earlier studies based on fMRI imaging [13]. The specially devised analyses of the HBOT imaging revealed that the improvements in the syndrome status went hand-in-hand with changes in the patterns of brain activity towards those of healthy subjects. More specifically, for the response patients, HBOT sessions led to reduction in brain activity in the somatosensory cortex and enhancement of the brain activity in the frontal, cingulate, medial temporal and cerebellar cortices.

### HBOT can rectify abnormal brain activity

Levels of pain sensations are determined by the sensory recording and higher level information processing (interpretation) in the brain. Evidence from previous studies suggests that the pain in fibromyalgia results primarily from abnormality in the function of pain processing pathways. In simple terms, it may be described as hyper-excitability of pain processing pathways and under-activity of inhibitory pain pathways in the brain, resulting in the affected individual experiencing pain. In the present study we found that HBOT can rectify chronically abnormal brain activity – decrease the activity of hyperactive regions (mainly posterior regions) and increase the activity of underactive regions (mainly frontal areas), in good agreement with the current knowledge regarding the brain’s response to pain.

More specifically, brain areas that are activated in response to pain are S1, S2 (BA 1, 2 and 3), insular cortex, anterior cingulate cortex (ACC), prefrontal cortex (PFC) and thalamus [44]. Anticipation of pain activates the anterior insula, ACC and PFC. It has also been shown that rostral ACC is activated in analgesia [45]. The effect of the ACC on pain processing is unclear, but one option that was suggested is that the release of the inhibitory neurotransmitter GABA and/or opioids reduces the excitability of ACC neurons that send descending innervations directly or indirectly to rostral ventromedial medulla neurons [45]. Consequently, this might cause less pain information to arrive from the spinal cord to the brain. Thus, the activation of the ACC and other frontal areas can prevent pain information from the spinal cord from reaching the brain and thus reduce activation in the rostral areas that receive this information.

### A quest for new understanding

Previous studies provided convincing evidence that HBOT could induce neuroplasticity leading to repair of chronically impaired brain functions and improved quality of life in post stroke patients and post mTBI patients with prolonged post concussion syndrome, even years after the brain insult [18–20]. HBOT can entail repair of brain damage resulting from stroke and TBI via an assortment of intricate mechanisms [18, 19, 46]. For example, it is known that HBOT can initiate vascular repair mechanism and improve cerebral vascular flow, induce regeneration of axonal white matter, stimulate axonal growth, promote blood-brain barrier integrity, and reduce inflammatory reactions as well as brain edema [24, 46–52]. At the cellular level, HBOT can improve cellular metabolism, reduce apoptosis, alleviate oxidative stress and increase levels of neurotrophins and nitric oxide through enhancement of mitochondrial function both in neurons and glial cells, and may even promote neurogenesis of endogenous neural stem cells [24, 46–52]. In FMS patients, glial cells might be hypothesized to play an integral role in the pathogenesis of central sensitization and chronic pain [53, 54]. Therefore, it is plausible that increasing oxygen concentration by HBOT can change the brain metabolism and glial function to rectify the FMS associated brain abnormal activity. It has already been demonstrated that exposure to hyperbaric oxygen induces a significant anti-inflammatory effect in different conditions and pathologies [21–24]. As such, it was also demonstrated that repetitive HBOT may attenuate pain by reducing production of glial cell inflammatory mediators [25, 26]. It can be informative to include, in future studies, additional modalities of brain monitoring such as EEG, fMRI and DTI, and to test the changes in brain response to pain stimulation in addition to assessments of changes in the base activity, as was done in this “proof of concept” study.

A supportive clinical observation for the notion that HBOT is indeed inducing neuroplasticity and is not merely “pain killer therapy” is the fact that a significant number of patients reported an increase or change in the pain sensation during the first 10–20 session. Consequent to this period of changed/increased pain sensation, patients reported a more comprehensive change beyond pain alleviation, including improvement in sleep characteristics and cognitive functions, more energy for daily tasks and improvement in general wellbeing. The symptom worsening during the first session might be related to HBOT-induced metabolic and circuitry changes in brain areas associated with pain interpretation. There might be intermediate stages in the HBOT-induced repair process of the abnormal metabolism and circuitry, during which the pain sensation can be further amplified before reaching normal metabolism and circuitry. However, currently this is only a plausible idea that calls for future studies. This intriguing phenomenon was not anticipated when the study was designed so it was not objectively evaluated; further studies are needed to investigate this newly discovered phenomenon.

### Study limitations

The study is subject to some limitations:

I. *Sample size*. Clearly, larger scale clinical trials are required to corroborate the findings presented here. In addition to statistical discussion on the sample size consideration in the study protocol (S1), another consideration for including sixty patients in a single clinical site, was our attempt to optimize between two contradicting constrains: 1. The need for diverse population treated in order to generalize the findings for a more heterogeneous group of patients. 2. The need to perform physiological evaluations for each of the participating patients, including repeated metabolic brain imaging. Further studies are needed in multiple clinical centers in order to evaluate the findings in larger heterogonous patient population.

II. *Diagnostic criteria*. As mentioned earlier, it is important to select proper diagnostic criteria for FMS. While the study started in 2010, it’s design and application were done earlier – well before the new criteria by Wolfe at al. [6] were proposed and accepted. Nevertheless, being aware of the limitations mentioned earlier that are associated with the 1990 ACR criteria, we quantitatively assessed the tender points and included additional functional impairment as well as psychological distress and quality of life evaluations. In retrospect, the assessment we used can be view as a combination of the 1990 and 2010 criteria. Yet, future studies might consider using the new, 2010 criteria.

III. *No double blinding*: While the division into two sub-groups was done randomly and so were the physiological evaluations and the SPECT assessments, the patients were not blinded because of the above mentioned placebo considerations. The non- blinded identity of the patients to the examiners may have an effect on the self assessment questionnaires (FIQ, SCL and SF-36). The agreement between the improvements as reflected in self assessment questionnaires and in pain thresholds and brain SPECT analyses, which was done in a blinded fashion, further substantiates the clinical findings. Moreover, the association between the anatomical locations of the changes in the brain metabolism, as demonstrated by the SPECT, and the clinical findings provides important validation of the evaluation.

IV. *Sham control*. There is an inherent difficulty in handling sham control in HBOT trials, as mentioned in the introduction and detailed below.

V. *Comparative studies*. Future studies are needed in order to compare HBOT with other therapeutic interventions used for FMS patients

### The sham control dilemma

Hyperbaric oxygen therapy includes two active ingredients: pressure and oxygen [46, 55]. The use of pressure is intended for increasing plasma oxygen, but pressure increase alone can have significant effects on the cellular level, in particular in organs that are pressure auto-regulated, such as the brain and kidneys [56–60]. More specifically, any increase in cranial pressure may have a significant effect on neurons, glial cells and the function of endothelial cells [56, 57, 60]. Put together, ample observations indicate that small increases in pressure, with normal or even reduced oxygen levels, cannot serve as placebo since they activates at least one of the two active ingredients of HBO_2_ therapy – pressure and level of tissue oxygen.

To engender the sensation of pressure, the chamber pressure must be 1.3 Atm abs or higher. This led several studies to mistakenly use HBO_2_ treatment at 1.3Atm with normal air as sham control, overlooking the fact that under such conditions the tissue oxygen level can increase by more than 50%, possibly resulting in significant physiological effects due to the elevated pressure and the tissue oxygenation. Therefore, such doses should be regarded as a dose-comparison study and not as sham control, as was correctly done by Mukherjee *et al*. who demonstrated that 1.3 Atm with normal air is effective in the treatment of children with CP [61].

As mentioned in the introduction, to circumvent the inherent sham control problem, we adopted the crossover approach that has already been successfully used to test the neurotherapeutic effects of HBOT [18, 19, 55, 62]. Clearly, the “placebo effect” is not fully resolved by the crossover approach, but what make the results sounder and suggest that the improvements are not likely to be a placebo effect are the following: 1. Only the responders showed significant changes in brain activity, and the changes rectified the known abnormality in brain activity of FMS patients. 2. Unexpectedly, in many of the patients, the symptoms worsened during the first 20 sessions.

### Looking ahead

Follow-up studies are needed in order to investigate the durability of the HBOT effects on FMS. It might be that some patients will need more HBOT sessions. The issue of how to optimize patient-specific protocols is an important subject for future research. We foresee that the future oxygen-pressure dose-response studies will have significant therapeutic implications. In particular, based on previous studies in mTBI patients, it can be anticipated that, for some patients, HBOT treatment at lower pressure and/or lower oxygen level can be effective. Our findings of changes in brain activity in the responsive patients indicate that non invasive monitoring, e.g. by EEG and fMRI, can help assess the response of the patients to the treatment and design person-specific dose-response adjustments.

## In conclusion

This study provides evidence that HBOT can improve quality of life and wellbeing of many FMS patients. It shows for the first time that HBOT can induce neuroplasticity and significantly rectify brain activity in pain related areas of FMS patients. Additional, studies are required to find the optimal dose-response curve and optimal time of treatment. The observation that pain characteristics may fluctuate, and even get worse during the first 10–20 sessions, before its resolution, deserves notice and future investigation. Since there is currently no solution for FMS patients, and since HBOT is obviously leading to significant improvement, it seems reasonable to let FMS patients benefit from HBOT, if possible, now rather than wait until future studies are completed.

## Supporting Information

S1 CONSORT Checklist CONSORT 2010 checklist. (PDF)

S1 Consent Form Informed consent form. (PDF)

S1 File Additional assessment of the within the crossover group. (DOCX)

S2 File Additional comparisons between groups. (DOCX)

S1 Protocol Clinical Study Protocol. (PDF)
