# Supplementary material for: Hyperbaric Oxygen Therapy Can Diminish Fibromyalgia Syndrome – Prospective Clinical Trial
Source: PLoS One. 2015 May 26;10(5):e0127012. doi: 10.1371/journal.pone.0127012 (PMC4444341; doi:10.1371/journal.pone.0127012)
Supplement: S1 CONSORT Checklist — (PDF) [file pone.0127012.s001.pdf]

SI3: CONSORT 2010 checklist of information to include when reporting a randomised trial

Section/Topic	Item No	Checklist item	Reported on page No
**Title and abstract**			
	1a	Identification as a randomised trial in the title	Article title
	1b	Structured summary of trial design, methods, results, and conclusions (for specific guidance see CONSORT for abstracts)	Article methods
**Introduction**			
Background and objectives	2a	Scientific background and explanation of rationale	Article introduction
	2b	Specific objectives or hypotheses	Article introduction
**Methods**			
Trial design	3a	Description of trial design (such as parallel, factorial) including allocation ratio	Article methods, Study protocol-SI1
	3b	Important changes to methods after trial commencement (such as eligibility criteria), with reasons	
Participants	4a	Eligibility criteria for participants	Article methods, Study protocol-SI1
	4b	Settings and locations where the data were collected	Article Methods
Interventions	5	The interventions for each group with sufficient details to allow replication, including how and when they were actually administered	Article methods, Study protocol-SI1
Outcomes	6a	Completely defined pre-specified primary and secondary outcome measures, including how and when they were assessed	Article methods, Study protocol-SI1
	6b	Any changes to trial outcomes after the trial commenced, with reasons	

Section/Topic	Item No	Checklist item	Reported on page No
Sample size	7a	How sample size was determined	Study protocol-SI1
	7b	When applicable, explanation of any interim analyses and stopping guidelines	
Randomisation:			
Sequence generation	8a	Method used to generate the random allocation sequence	Article methods, Study protocol-SI1
	8b	Type of randomisation; details of any restriction (such as blocking and block size)	Article methods, Study protocol-SI1
Allocation concealment mechanism	9	Mechanism used to implement the random allocation sequence (such as sequentially numbered containers), describing any steps taken to conceal the sequence until interventions were assigned	Article methods, Study protocol-SI1
Implementation	10	Who generated the random allocation sequence, who enrolled participants, and who assigned participants to interventions	Article: methods, Study protocol-SI1
Blinding	11a	If done, who was blinded after assignment to interventions (for example, participants, care providers, those assessing outcomes) and how	Article methods, Study protocol-SI1
	11b	If relevant, description of the similarity of interventions	
Statistical methods	12a	Statistical methods used to compare groups for primary and secondary outcomes	Article methods
	12b	Methods for additional analyses, such as subgroup analyses and adjusted analyses	Article methods
**Results**			
Participant flow (a diagram is strongly recommended)	13a	For each group, the numbers of participants who were randomly assigned, received intended treatment, and were analysed for the primary outcome	Article results
	13b	For each group, losses and exclusions after randomisation, together with reasons	Article results
Recruitment	14a	Dates defining the periods of recruitment and follow-up	Article results
	14b	Why the trial ended or was stopped	Sample size
Baseline data	15	A table showing baseline demographic and clinical characteristics for each group	Article results
Numbers analysed	16	For each group, number of participants (denominator) included in each analysis and whether the analysis was by original assigned groups	Article results
Outcomes and	17a	For each primary and secondary outcome, results for each group, and the estimated effect size and its	Article results

estimation		precision (such as 95% confidence interval)	
	17b	For binary outcomes, presentation of both absolute and relative effect sizes is recommended	
Ancillary analyses	18	Results of any other analyses performed, including subgroup analyses and adjusted analyses, distinguishing pre-specified from exploratory	Article results
Harms	19	All important harms or unintended effects in each group (for specific guidance see CONSORT for harms)	Article results
**Discussion**			
Limitations	20	Trial limitations, addressing sources of potential bias, imprecision, and, if relevant, multiplicity of analyses	Article discussion
Generalisability	21	Generalisability (external validity, applicability) of the trial findings	Article discussion
Interpretation	22	Interpretation consistent with results, balancing benefits and harms, and considering other relevant evidence	Article discussion
**Other information**			
Registration	23	Registration number and name of trial registry	Article 1st page
Protocol	24	Where the full trial protocol can be accessed, if available	Study protocol-S1
Funding	25	Sources of funding and other support (such as supply of drugs), role of funders	Article 1st page Financial Disclosure
