# Supplementary material for: Hyperbaric Oxygen Therapy Can Diminish Fibromyalgia Syndrome – Prospective Clinical Trial
Source: PLoS One. 2015 May 26;10(5):e0127012. doi: 10.1371/journal.pone.0127012 (PMC4444341; doi:10.1371/journal.pone.0127012)
Supplement: S1 Consent Form — (PDF) [file pone.0127012.s002.pdf]

טופס 2 א' - עמוד 1 מתוך 3

Informed consent form

Ethics Committee request number

I, the undersigned:

Full name:	
ID number:	
Address:	zip code:

A) Hereby certify that I agree to participate in a clinical trial as detailed in this document.

B) Hereby certify that I am not currently participating in any other clinical trial involving the use of any research product, and that I shall not participate in any other clinical trial involving the use of another research product throughout this trial period.

C) Hereby acknowledge to have been explained to me by

Name of informing investigator

1. That the principal investigator, Dr. Shai Efrati, has been granted approval by the head of the medical institution where the trial is to be conducted to perform the clinical trial in human subjects, as stated in the Public Health Regulations (clinical trials on human subjects) 1980, (hereby the clinical trial).
2. That the principal investigator is the initiator of the trial.
3. That the clinical trial is conducted to test hyperbaric treatment for patients suffering from fibromyalgia.
4. That I am free to withdraw my consent and to discontinue participation at any time with no impairment to my right to receive the commonly accepted treatment.
5. That if asked to fill a questionnaire, I may refrain from answering all or part of the questions in the questionnaire.
6. That I'm guaranteed that my identity will be kept confidential by all those involved in the trial and will not be disclosed in any publication, including scientific ones.

7. That the medical institution has arranged for proper insurance of the investigators, medical doctors and medical staff involved in the trial, covering claims that may be filed by trial participants and/or third party claims related to the clinical trial, during the trial or after.

8. that if necessary, by the principal investigator recommendation, I shall be able to continue receiving the research product free of charge for up to three years after the end of the clinical trial, if there is no alternative medical treatment for me. This, provided that the product has not been approved for use in Israel for the indication requested in the trial and is not available from the HMO in which I'm insured.

The decision regarding continuation of the research product is to be given by the Institutional Ethics committee, subject to the existence of a treatment and monitoring plan.

9. That I am guaranteed readiness to answer any questions I may have later and the option to consult with another party (e.g. family physician, family members, etc.), regarding my decision to enroll or continue my participation in the clinical trial.

10. That if I become pregnant during the clinical trial, I shall be advised (by the investigator) regarding possible outcomes to the fetus and the pregnancy, including the possibility of terminating the pregnancy.

11. That I can contact Dr. Shai Efrati (Tel. # 0577346364) any time, 24 hrs a day, about any problem relating to the clinical trial.

D) I hereby declare that I received detailed information about the clinical trial as listed below:

1. The purpose of the study: to evaluate the effect of treatment with high-pressure oxygen on patients suffering from fibromyalgia.

2. The estimated number of participants in the clinical trial is 60.

3. The expected duration of the trial: 8 weeks treatment in hyperbaric chamber and about 4 weeks of follow-up including pre and post treatment evaluations.

4. Methods: description of the research product, description of the various procedures during the trial (treatment and follow-up), while making a clear distinction between the research procedures and the usual medical procedures; indication of the participant's chances of receiving each of the treatments offered in the trial (including placebo, if included):

After admission, participants will be divided into two groups, one of which will receive hyperbaric

treatment at the beginning of the trial and the other after about two months.

The hyperbaric treatment is given in the Hyperbaric Medicine unit at Assaf Harofe hospital. Once the patient is in the chamber, the pressure is raised to 2 atmospheres and 100% oxygen is introduced into the chamber. Treatment sessions are one and a half hours long and are given five days a week, for eight weeks – altogether 40 sessions.

The participants are to be evaluated upon inclusion, after two months, and at the end of the study after four months. Evaluations include tender point count, functional impairment, Symptoms severity (SCL-90 questionnaire) and Quality of life (SF-36 questionnaire). In addition at each evaluation point brain SPECT will also be done. Participants in the treated group are evaluated twice – at baseline and after 2 months of HBOT treatment. Participants in the cross group are evaluated three times: at baseline, after 2 months control period of no treatment, and after consequent 2 months of HBOT sessions. The post-HBOT evaluations will be performed more than 1 week (1-4 weeks) after the end of the HBOT protocol.

During treatment in the hyperbaric chamber, the 2 atm pressure is felt mostly in the ears and it is necessary to equalize pressure by shutting the nose and pressing in the mouth toward the ears. Other ways to equalize are by drinking water or swallowing. If one fails to equalize, damage to the eardrum may occur in various degrees, from redness and local edema to a puncture.

Another organ that may be harmed by the pressure is the lung. To address this, each participant will undergo chest X-ray. If pathology is found, raising the risk in high pressure, treatment will not be given.

5. Potential benefits to participants.

Current knowledge supports the notion that hyperbaric treatment may improve the symptoms of fibromyalgia. The study participants will receive the hyperbaric treatment and full evaluations at no cost.

6. Known risks and/or discomfort that participants may incur.

If the trial bears risk to the participant – an explanation of the medical treatment s/he will receive in case of harm and by whom.

As explained above, during treatment air pressure is raised to 2 atm. The high pressure can cause damage to the eardrum, which may be expressed as pain, redness, local edema, and even a puncture. To avoid this, it is necessary to equalize pressure as explained.

High pressure may also cause damage to the lung tissue and result in pneumothorax, where air leaving the lungs is trapped between the lung tissue and the chest wall. Thus, participants who reveal lung pathology in chest X-ray will not be given hyperbaric treatment.

High oxygen levels may cause oxygen toxicity, which may lead to spasm. That is why exposure

time is limited, depending on the pressure in the chamber.

7. Circumstances in which participation may be terminated by the investigator or initiator:
If the patient does not arrive for treatment and medical testing beyond reasonable;
If side effects of any kind appear that the doctors assess as possibly related to the hyperbaric treatment.
The patient may terminate her/his participation at any time during the study.

8. The investigator will provide to the participant information regarding possible medical consequences of her/his decision to leave the study before the end.

9. Explanation about alternative treatments and their advantages and disadvantages, if any, to the participant.

Other treatment modalities that may help include neurological rehabilitation by physical therapy, occupational therapy, biofeedback and physiotherapy of any kind. These therapies are not prohibited and participants may continue with them in addition to hyperbaric treatments. However, any change or additional treatment should be reported to the investigator.
10. Any other relevant information (as related by the study initiator):

E) I hereby declare that I gave my consent of my free will and that I understand all that is said above. I was given a copy of this informed consent form, dated and signed appropriately.

F) By signing this consent form, I give permission to the initiator of the clinical trial, to the institutional Ethics Committee, to the auditor of the nedical institute and to the Ministry of Health direct access to my medical records, for the purpose of verifying the clinical methods and data/ This access to my medical records will be done in complete confidentiality, according to the rules and regulations of confidentiality.

G) In case that the clinical trial will involve certain medical tests or services, I hereby declare that I know and agree that my participation in the clinical study will be disclosed to my personal physician at the HMO/health care service where I'm insured.
I know that the HMO/health care service will not use this information for any purpose other than medical

treatment and follow-up.

Name of study participant	Signature of study participant	Date

If needed

Name of independent witness	ID number	Signature of witness	Date

<u>Investigator declaration</u>

This consent was received by me, after I had informed and explained to the participant all of the above and verified that all my explanations were understood by her/him.

Name of informing investigator	signature	Date
