# Supplementary material for: Hyperbaric Oxygen Therapy Can Diminish Fibromyalgia Syndrome – Prospective Clinical Trial
Source: PLoS One. 2015 May 26;10(5):e0127012. doi: 10.1371/journal.pone.0127012 (PMC4444341; doi:10.1371/journal.pone.0127012)

**Hyperbaric Oxygen Therapy Can Diminish Fibromyalgia Syndrome – Prospective Clinical Trial**

**S1 File: Additional assessment of the within the crossover group comparison of the relative changes in the FMS symptoms between the control period and the treatment period**

In the main part of the article we presented the relative changes in the values of these parameters during the control period of the crossover group, during the treatment period of the crossover group and during the treatment period of the treated group. The results for the relative changes in the Dolorimeter pain threshold and in the number of tender points are shown in Figure 3. The results for the relative changes in the FIQ score, in the SL-90 score and in the SF-36 are shown in Figure 5.

Here we present an additional within-group comparison for the crossover group. The motivation is to test validate the observed improvements in the patients of the crossover group happened during the treatment period. For this we compared the changes in the physiological parameters during the control + treatment periods (referred to in the main text as the combined period) with those during the treatment period. The results for the relative changes in the Dolorimeter pain threshold and in the number of tender points are shown in Figure A. The results for the relative changes in the FIQ score, in the SL-90 score and in the SF-36 are shown in Figure B.

**Figure A:** Assessments of the mean relative changes in the pain level. 1) The mean relative change and standard errors in the Dolorimeter thresholds for the crossover group during the HBOT treatment period (blue) and during the combined period (orange). 2) The same as (1) for the number of tender points.

**1**  **2**


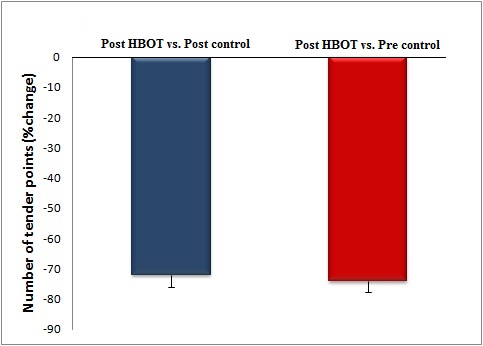

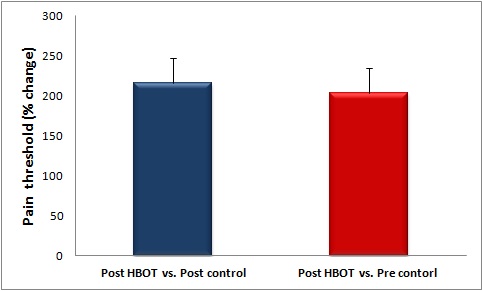


**Figure B:** Assessments of the mean relative changes in the FIQ score (1), in the SL-90 score (2) and in the SF-36 score (3). The mean relative change and standard errors in these measures during the treatment period and during the combined period are presented using the same color code as in Figure A.

**1 2**


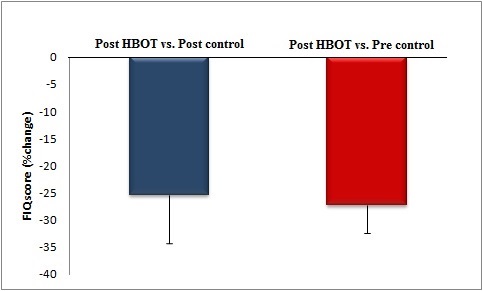

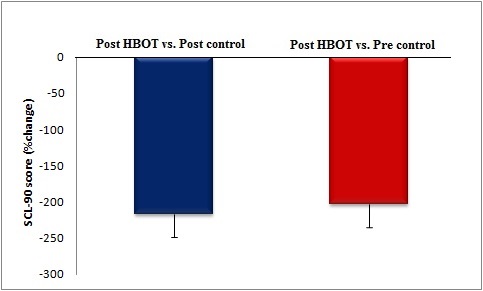


**3**


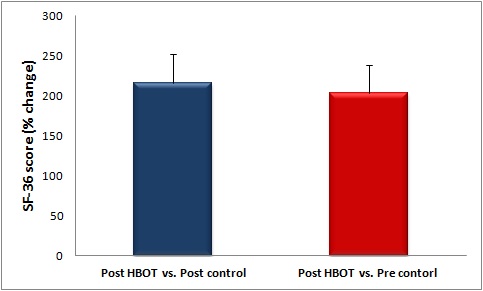

Supplement: S1 File — (DOCX) [file pone.0127012.s003.docx]
