# Supplementary material for: Hyperbaric Oxygen Therapy Can Diminish Fibromyalgia Syndrome – Prospective Clinical Trial
Source: PLoS One. 2015 May 26;10(5):e0127012. doi: 10.1371/journal.pone.0127012 (PMC4444341; doi:10.1371/journal.pone.0127012)
Supplement: S2 File — (DOCX) [file pone.0127012.s004.docx]

**Hyperbaric Oxygen Therapy Can Diminish Fibromyalgia Syndrome - Randomized Prospective Trial**

**S2 File: Additional comparisons between groups**

In this SI we present additional comparisons between the tested groups. We constructed similar histograms as the ones presented in figures 6 and 7A in the main text, but while in the main text the comparison was between the response group after HBOT period and the crossover group following the control period, in this SI we performed comparisons between different types of groups.

**Control group compared to treatment group (all 48 patients):** Comparison between the changes in SPECT perfusion during control period and during treatment period of all patients (without separation between the response and the nonresponse groups) is presented in figure A.


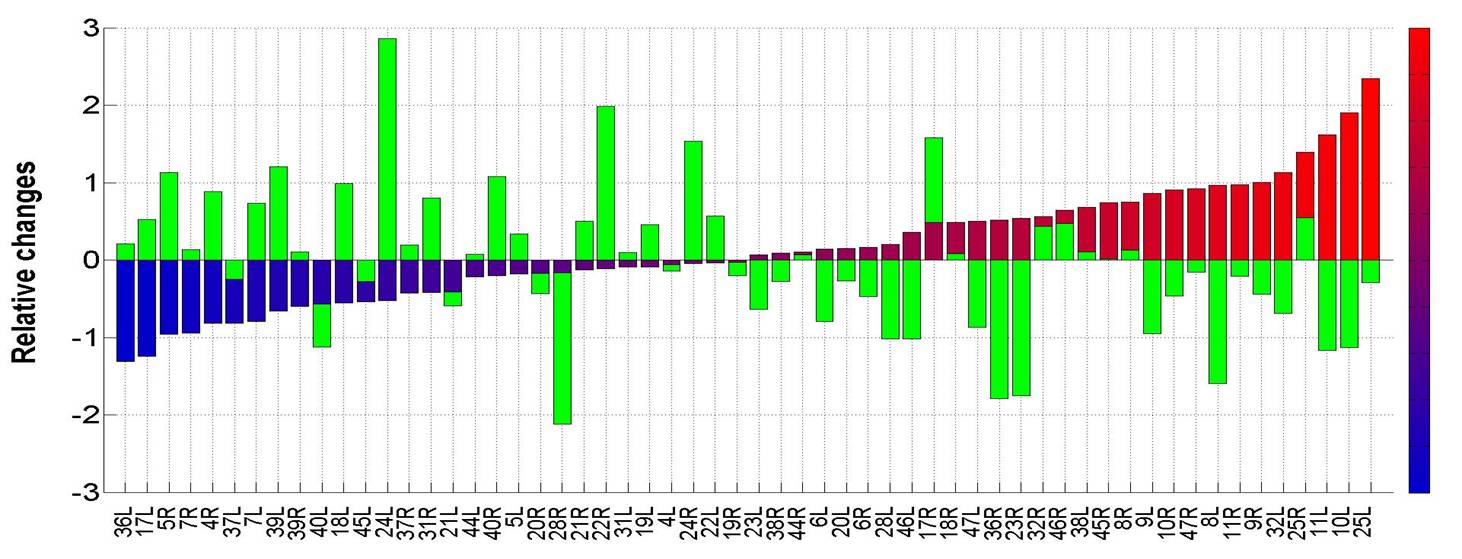


**Figure A:** BA histogram of mean relative changes. The Y-axis shows the mean relative change <R­_change_>(n) for the Brodmann area indicated in the X-axis. The figure is similar to Figure 6 in the main text, only the light blue to light red bars correspond to the group of all patients after HBOT treatment and not only to the response group. The green bars correspond to the mean relative changes of the patients in the crossover group following the control period.

In Figure B we show similar histogram to the aforementioned one but in which we normalized the mean relative changes of each BA (n) by its corresponding significance index I_σ_(n) as is defined and explained in the Methods section in the main text.

**
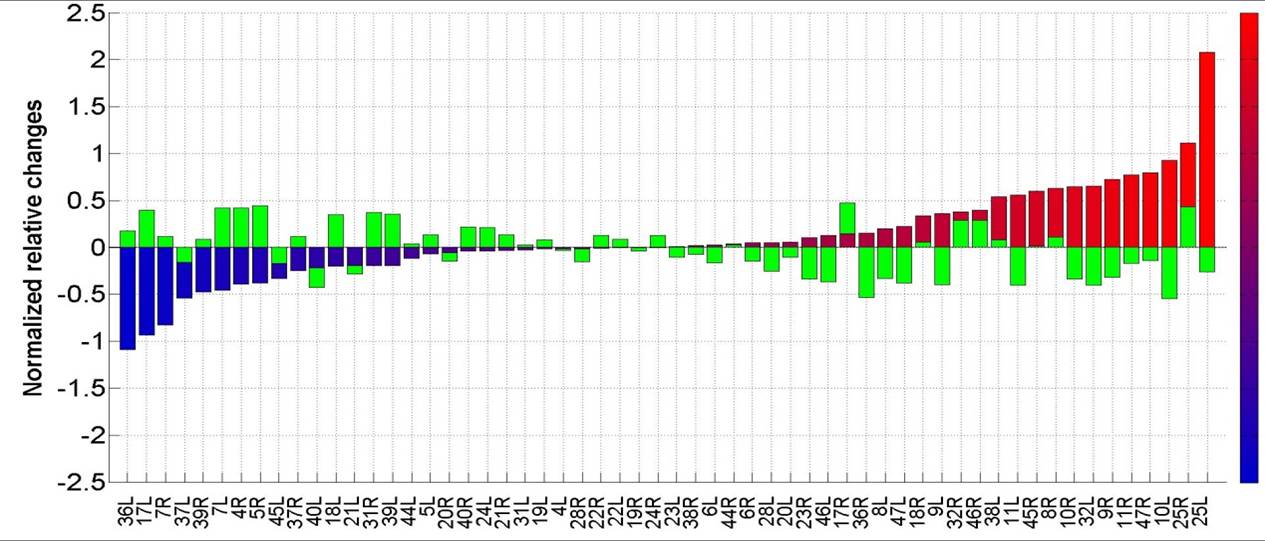
**

**Figure B:** The effect of significance index normalization: Normalized BA histogram of mean relative changes. The figure is similar to Figure 1 but the Y-axis is for the normalized values, that is for I_σ_(n)* <R­_change_>(n) and not for <R­_change_>(n) that are used in Figure A. The color code is the same as the one used in Figure A.

**Response group compared to Nonresponse group:** As is explained in the main text, the patients were classified to the response and nonresponse groups based on the changes in their physiological symptoms following the treatment (number of tender points and the level of threshold pressure). 41 out of 48 patients were classified as responders (response group) while the 7 other patients were classified as non-responders and were assigned to a nonresponse group. Comparison between the two groups is shown in Figures C and D compare between the groups.

**
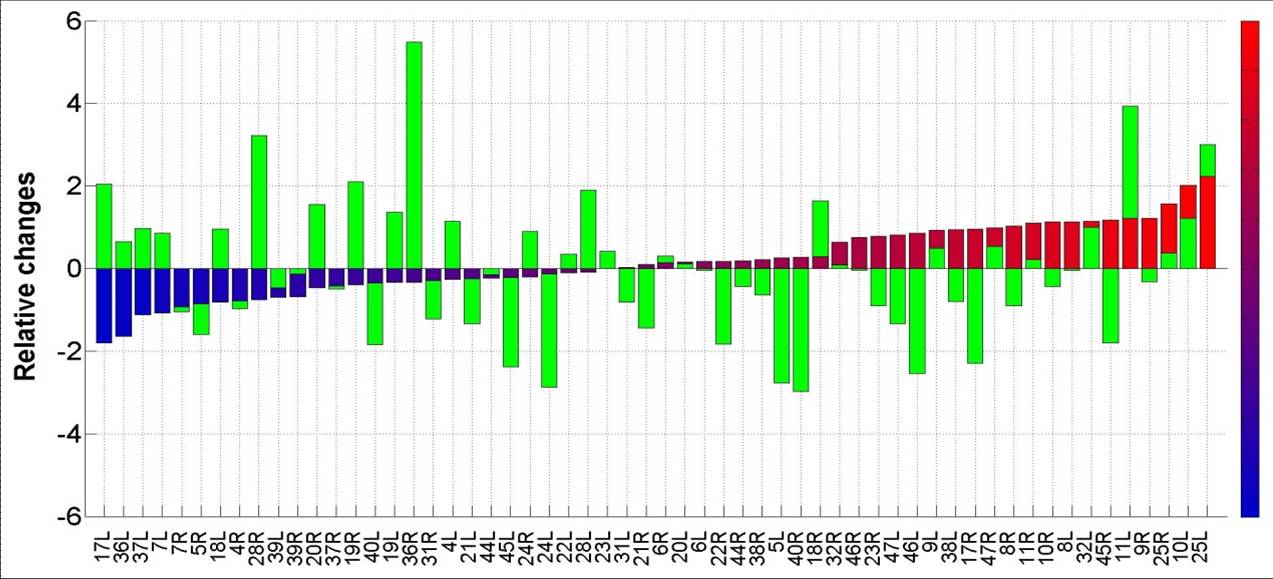
**

**Figure C:** BA histogram of mean relative changes. The figure compares two groups of patients: The light blue to light red bars present the response group after HBOT treatment and the green bars correspond to nonresponse group after HBOT treatment. The axes correspond to the same explanation as in figure A.

**
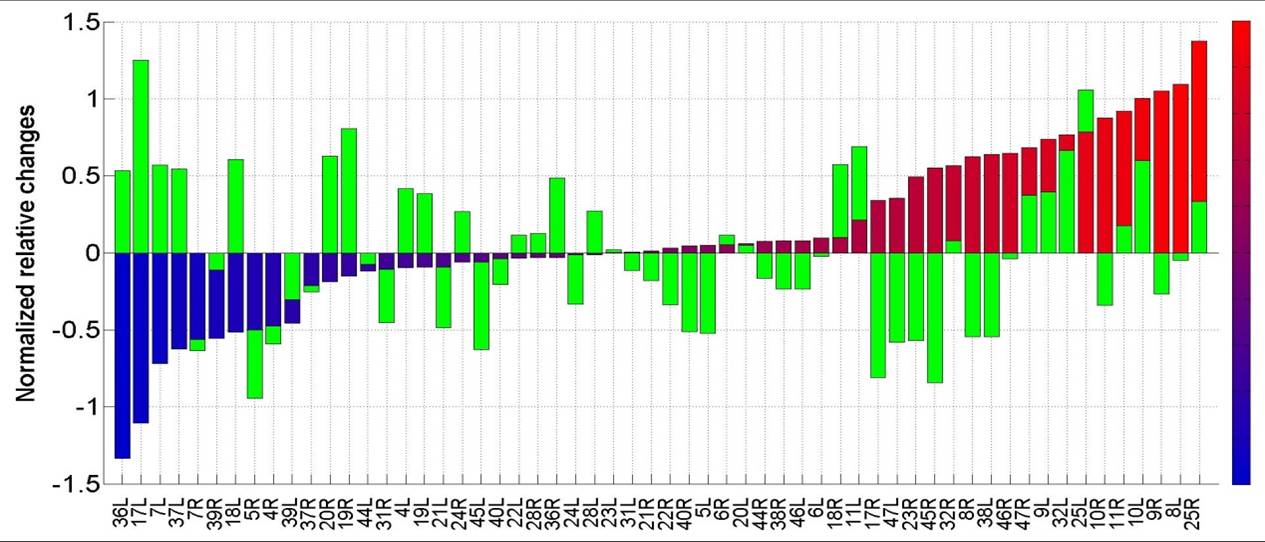
**

**Figure D:** The effect of significance index normalization: Normalized BA histogram of mean relative changes. The figure is similar to Figure C only light blue to light red bars correspond to the response group after HBOT treatment and the green bars correspond to the mean relative changes of the nonresponse group after HBOT treatment.
