# Supplementary material for: Hyperbaric Oxygen Therapy Can Diminish Fibromyalgia Syndrome – Prospective Clinical Trial
Source: PLoS One. 2015 May 26;10(5):e0127012. doi: 10.1371/journal.pone.0127012 (PMC4444341; doi:10.1371/journal.pone.0127012)
Supplement: S1 Protocol — (PDF) [file pone.0127012.s005.pdf]

# S1: Clinical Study Protocol

---

| | |
|---|---|
| **Study Title:** | **Hyperbaric Oxygen Therapy for Fibromyalgia - Randomized, Prospective trial** |
| **Protocol Number:** | HBOT-Fib -01 Version 3.0 |
| **Investigational Product:** | Hyperbaric Oxygen Therapy, 2ATM, 100% oxygen<br>Hyperbaric chamber, Multiplace at Assaf-Harofeh Medical Center, Israel |
| **Primary Investigator:** | Dr. Shai Efrati<br>Fax: +972-(0)8-920-4989<br>Phone: +972-(0)8-977-9393/5<br>Cell : +972-549-212-866<br>Email : efratishai@013.net |
| **Study Director:** | Yair Bechor<br>Fax: +972-(0)8-920-4989<br>Cell: +972-577-345-624 |

## Content


**Pages**

Background………………………………………………………………………....3-5

Objectives……………………………………………………………………...5-6

Study design…………………………………………………………………......6-10

Inclusion criteria………………………………………………………..6

Exclusion criteria………………………………………………………6-7

Study protocol……………………………………………………….....7-10

Administration and regulation…………………………………………....11

Informed Consent………………………………………………………11

Confidentiality ……………………………………………………………11

Study Files………………………………………………………….…..11

Statistical considerations...………………………………………………......12-13

Safety Analysis Set…………………………………………….………12

Primary Efficacy Analysis Set……………………………………….12

Sample Size Considerations…………………………………….…....12-13

Randomization ………………………………………………………….…….13-14

Adverse Events…………………………………………………………………..14-17

References ……………………………………………………………………..18

## Background

Fibromyalgia is considered to be a disorder of pain regulation, classified often under the term central sensitization. Fibromyalgia shares several features with other common pain disorders that are considered to be more central rather than peripheral pain conditions, such as migraine, tension headaches, temporomandibular joint disorder, and irritable bowel syndrome; these features include common genetic and central nervous system pain processing characteristics.

Diagnostic classification criteria were proposed, evaluated, and then validated. Using such criteria, fibromyalgia is now considered to be the most common cause of generalized, musculoskeletal pain in women between ages of 20 and 55 years; in the United States and in other countries, the prevalence is approximately 2 percent and increases with age. Fibromyalgia is six times more common in females in reports from specialty clinics, although the female predominance is not as striking in the community. More than 40 percent of patients referred to a tertiary pain clinic meet the diagnostic criteria for fibromyalgia.

**Symptoms** - The cardinal manifestation of fibromyalgia is widespread musculoskeletal pain, involving both sides of the body and above and below the waist. However, the pain may initially be localized, often in the neck and shoulders. Common patient descriptions include "I feel as if I hurt all over", or "it feels as if I always have the flu." Patients typically describe pain predominantly throughout the muscles, but often state that their joints hurt, and sometimes describe joint swelling, although synovitis is not present on examination.

- Patients also often report paresthesias, including numbness, tingling, burning, or creeping or crawling sensations, especially in both arms and both legs.

- The other universal symptom of FM is fatigue. This is especially notable when arising from sleep, but is also marked in the mid-afternoon.
- Depression and/or anxiety are present in 30 to 50 percent of patients at the time of diagnosis.
- Headaches are present in more than 50 percent and include migraine and muscular (tension) types.
- Patients also may have a variety of poorly understood pain symptoms, including abdominal and chest wall pain and symptoms suggestive of irritable bowel syndrome, pelvic pain and bladder symptoms of frequency and urgency suggestive of the interstitial cystitis/painful bladder syndrome (formerly female urethral syndrome).
- Other common complaints include ocular dryness, multiple chemical sensitivity and "allergic" symptoms, palpitations, dyspnea, vulvodynia, dysmenorrhea, sexual dysfunction, weight fluctuations, night sweats, dysphagia, dysgeusia, and orthostatic intolerance.

**Hyperbaric oxygen therapy and Fibromyalgia**

Hyperbarric Oxeygen Therapy (HBOT) is a well known, generally safe therapeutic modality, which has been implemented in a variety of clinical conditions. The straightforward aim of HBOT is to increase the oxygen content of blood, through application of the combination of hyperbaric pressure and increase oxygen content. While the beneficial effects of HBOT on conditions such as peripheral ischemia seem self-evident, the effect on the CNS is far more complex. Previous research has indicated that HBOT may induce neuroplasticity in different

brain injury models. This may be achieved by various effects on brain tissue, including an improvement in cerebral vascular flow, as well as enhanced cellular and vascular repair. One possible mechanism explaining the neuroplasticity potential of HBOT relates to the effect on the mitochondrial function of Glia cells, a cell population which is postulated to play an integral role in the pathogenesis of central sensitization and chronic pain. Breathing oxygen under hyperbaric conditions has the capacity to significantly increase brain oxygen tension, increasing tissue oxygenation and oxygen delivery into Glial mitochondria.

Recently, in a pilot study, Yildiz et al have reported a significant decrease in tender points and pain VAS among FMS patients treated with HBOT. In this pilot study the HBOT protocol (15 sessions of 2.4 ATA) is consider to be short and even thought they report pain sensation reduction they did not evaluated the effect of HBOT on the daily living and the quality of life of the participated patients. Another, interesting evaluation missing from their study is the effect of HBOT on the control group after the control period.

**Objectives**

The aim of the current study is to evaluate, the utility of HBOT as a therapeutic modality for FMS.

The primary end point of the study was pain evaluation (as detailed below). Secondary end points included:

- Functional impairment (evaluated by the Fibromyalgia Impact Questionnaire- FIQ)[1],
- Symptoms severity (evaluated by the SCL-90 questionnaire)[2]
- Quality of life evaluation using the SF-36[3, 4]

- Metabolic imaging of the brain by SPECT analysis

The safety of the HBOT in this FM population will also be evaluated and any adverse event will be recorded.

**Study design**

A prospective, randomized, control-crossed over trial.

The study will be done in the hyperbaric chamber and in the research and development unit of Assaf Harofeh Medical Center, Israel.

Data will include evaluation of level of pain by a blinded rheumatologist (tender point count and dolorimetry) extensive evaluation of parameters relating to daily function such as presence of widespread pain, fatigue, physical and social function, psychological distress and quality of life. In addition brain metabolism will be evaluated by brain SPECT.

**Inclusion criteria**

Patients suffering from FMS ages 21- 67 years. Patients should be diagnosed with fibromyalgia at least 2 years prior to their inclusion.

The diagnosis of fibromyalgia should be based on the following two criteria: (1) Symptoms of widespread pain, occurring both above and below the waist and affecting both the right and left sides of the body; (2) Physical findings of at least 11 of 18 tender points.

**Exclusion criteria**

Patients will be excluded if they will have one of the following criteria:

- Had been treated with HBOT for any other reason prior to their inclusion
- Have any other indication for HBOT;
- Chest pathology incompatible with pressure changes;
- Inner ear disease;
- Patients suffering from claustrophobia;
- Inability to sign written informed consent.
- Smoking patients are not allowed to smoke during the study and if they could not comply with this demand they will be excluded.
- Inability to sign informed consent

***Study protocol***

The protocol will be approved by the local Helsinki committee and all patients will signed written informed consent before their inclusion. The study will be conducted in the hyperbaric and research units of Assaf-Harofeh Medical Center, Israel.

After providing written informed consent, patients are scheduled for baseline evaluation. Recruited patients will be randomized into two groups (1:1 randomization): a ***treated*** group and a ***cross*** group. Study endpoints includes tender point count, functional impairment (Fibromyalgia Impact Questionnaire- FIQ)[1], Symptoms severity (SCL-90 questionnaire)[2], Quality of life (SF-36 questionnaire)[3, 4] and brain SPECT. Patients in the ***treated*** group are evaluated twice – at baseline and after 2 months of HBOT treatment. Patients in the ***cross*** group are evaluated three times: at baseline, after 2 months control period of no treatment, and after consequent 2 months

of HBOT sessions. The post-HBOT evaluations will be performed more than 1 week (1-4 weeks) after the end of the HBOT protocol.

The following HBOT protocol will practiced: 40 daily sessions, 5 days/week, 90 minutes each, 100% oxygen at 2ATA. The total expected duration of HBOT is 2 months.

***Tender point evaluation***

At each time point of evaluation, participants will underwent tender point assessment by a rheumatologist, who will be blinded to group assignment. Tenderness will be assessed manually and with a dolorimeter. A count of 18 tender points at nine symmetrical sites will be performed by thumb palpation. The amount of manual pressure applied over a tender point is about 4 kg/cm2 (tested with a dolorimeter). Thirteen point sites (nine tender point sites and four control sites) will be further studied using a dolorimeter. Threshold of tenderness will be measured using a Chatillon dolorimeter, model 719-20, which has a maximum scale of 9 kg, with a neoprene stopper footplate with a diameter of 1.4 cm (Pain Diagnostics & Thermography Inc.,New York, USA)[4]. All dolorimeter measurements of the 13 point sites, as well as a total point count, will done by one rheumatologist, who will be blinded to patient group.

***Functional impairment***

The validated Hebrew version of the Fibromyalgia Impact Questionnaire (FIQ)[1] will be used to evaluate the level of functional impairment. The first part of the FIQ focuses on the patient’s ability to perform daily tasks (i.e. driving, cleaning, gardening, etc.) and contains 10 items with responses ranked 0 to 3, where 0=“always able”, and 3=“never able”. The item scores will be

normalized to range from 0 to 10 for uniformity, with 10 representing worst physical function. The mean of the items yields a single physical function score.

***Symptoms severity***

The Symptom Check List (SCL-90)[2] will be used to examine the level of psychological distress. This questionnaire consists of 90 items measuring 9 clinical subscales. It was developed as a measure of general psychiatric symptom severity and as a descriptive measure of psychopathology and has been found to be useful in the assessment of neurotic symptoms. The clinical subscales are: somatisation (12 items), obsession- compulsion (10 items), interpersonal sensitivity (9 items), depression (13 items) and anxiety (10 items), hostility (6 items), phobic anxiety (7 items), paranoid ideation (6 items) and psychoticism (10 items). The scale has been extensively used and validated in Hebrew. Subjects are required to rate how much they are bothered by specific complaints on a 5-point Likert scale from 0="never" to 4="frequently". A higher score indicates more distress. SCL-90. Internal consistency of the SCL-90 questionnaire was computed using internal consistency Cronbach alpha measure.

***Quality of life***

Quality of life (QoL) will be assessed by SF-36[3, 4]. This is a health-related profile of QoL that contains 36 items and measures health status across three domains: functional status, well-being and overall evaluation of health. The Hebrew translation of the SF-36 was validated in an adult general population, and our group has used it on patients with widespread pain, with and without FMS[5] . The SF-36 contains eight scales: physical functioning, social functioning, and role limitations attributable to physical and emotional problems, mental health, vitality, bodily pain

and general health. Each scale generates a score from 0 to 100, with a high score indicating better health and less body pain. Internal consistency of the SF-36 questionnaire was computed using internal consistency Cronbach alpha measure.

The sense of coherence questionnaire will be used in order to evaluate the way of managing stress and utilizing internal resources[6]. This instrument has previously been translated into Hebrew and utilized in Israeli patients suffering from FMS and IBS[7].

**Brain Functional Imaging- SPECT**

Brain single photon emission computed tomography (SPECT) will be conducted with 925–1,110 MBq (25–30 mCi) of technetium-99m-methyl-cysteinate-dimmer (Tc-99m-ECD) at 40–60 min post injection using a dual detector gamma camera (ECAM or Symbia T, Siemens Medical Systems) equipped with high resolution collimators. Data was acquired in 3-degree steps and reconstructed iteratively with Chang method ($\mu$=0.12/cm) attenuation correction.

Regional cerebral blood flow change analysis will be conducted by fusing pre- and post-treatment studies that were normalized to median brain activity. SPECT images will be reoriented into Talairach space using NeuroGam (Segami Corporation) for identification of Brodmann cortical areas and in order to compute the mean perfusion in each Brodmann area (BA). All SPECT analyses will be done while blinded to the laboratory and clinical data.

## Administration and regulation

### Informed Consent

The investigator will obtain written informed consent from the patient participates in this study after adequate explanation of the aims, methods, objectives, and potential hazards of the study and prior to undertaking any study-related procedures.  The investigator must utilize a consent form for documenting written informed consent.  Informed consent will be appropriately signed and dated by the patient or the subject's legally authorized representative and the person obtaining consent.

### Confidentiality

Subjects' anonymity will be strictly maintained and that their identities are protected from unauthorized parties. The information is not to be disclosed to any third party (except for medical stuff or employees or agents directly involved in the conduct of the study or as required by law).

### Study Files

The medical records will be maintained adequately to enable good data storage and latter on management.  Subject clinical source documents would include (although not, limited to) the following: subject hospital/clinic/ hyperbaric unit records, physician's and nurse's notes, appointment book, original laboratory reports, electroencephalogram (EEG), X-ray, SPECTs, CT and special assessment reports, consultant letters, screening and enrollment log  etc.

## Statistical Considerations

### *Analysis Sets*

### Safety Analysis Set

The safety analysis set will consist of all subjects for whom the study treatment was initiated.

### Primary Efficacy Analysis Set

In this trial will be measured the co-primary endpoint parameters: *improvement in tender points count, dolorimeter threshold of 9 tender sites,* following HBOT treatment for Fibromyagia patients suffering chronic pain.

The primary efficacy analysis evaluation will include all subjects who completed the HBOT treatment or the control period; had no major protocol violations, and for whom there is a valid *tender points count, dolorimeter threshold of 9 tender sites* following 2 months of HBOT treatment.

### Sample Size Considerations

Presentation of sample size is based on achieving 80% power overall to demonstrate that improvement rate in *dolorimeter threshold of the tender sites* is at least 0.62 (these improvements were based on the improvements reported by Yildiz et al. 2004). We used the threshold of tender sites as a criterion for sample size since this was the smaller effect. An improvement rate of at least 87% in the threshold of the tender sites in the treatment group (comparing to baseline, prior to HBOT treatment) would be an appropriate clinical target for HBOT treatment success and of sufficient interest to encourage further investigation of HBOT treatment for Fibromyalgia patients.

Sample size was based on the assumption that exposure to the dolorimeter evaluation (at baseline) without any additional training might induce up to 8% (0.06 dolorimeter change) improvement in the second dolorimeter evaluation (following treatment) (again, based on Yildiz et al.). Assuming a true success rate of 87% a sample of N = 31 will provide 80% power to show that HBOT treatment induces at least 87% improvement on *dolorimeter threshold of the tender sites*. This is based on a power analysis using the normal approximation for the binomial, with one-sided Alpha=0.05.

**Randomization**

Since the diversity of the patients included in the study, after signing the informed consent is expected to be relatively high, no stratifies criteria will be used. Patients were randomized in 1:1 fashion to either treatment or controlled-cross group.

There is no placebo in the cross group during the control period. The only way to give “placebo” of HBOT, is to bring the patients to the hyperbaric chamber and to increase the environmental pressure- in a way the patients will “feel the hyperbaric pressure” in their ears. However, Henry’s law states: “the amount of a given gas dissolved in a given type and volume of liquid is directly proportional to the pressure of that gas in equilibrium with that liquid”. Thus, hyperbaric environment significantly increases the dissolved oxygen pressure even if a person holding his breath[8]. Moreover, the alternative of hosting the patients in the hyperbaric chamber without any pressure increase cannot serve as a real placebo since the patients will not feel the pressure. Accordingly, the only real “placebo” could be archived by increasing the pressure and reducing the percentages of the oxygen but that holds significant safety consideration.

From the ethical point of view, it was deemed unethical to “treat” the patients (40 sessions in the hyperbaric chamber) while they actually know that it is a placebo. In order to somewhat compensate for this limitation, the control was a cross group and after the 2nd evaluation at the end of a control period they were crossed to HBOT. The cross for treatment enables intra-group efficacy evaluation in both treatment and control group.

Since the patients knew they were not receiving HBOT during the control period they were not blinded with regard to the treatment arm. Accordingly the questioner evaluations will not be blinded. However, in the current clinical setting, the physician doing the pain evaluation was done completely blinded by the physician.

**Adverse Events**

An adverse event (AE) is any untoward medical occurrence in a clinical investigation subject administered a medicinal product and which does not necessarily have a causal relationship with this treatment. An AE can therefore be any unfavorable and unintended sign, symptom, or disease temporally associated with the use of a medicinal product, whether or not considered related to the medicinal product. Pre-existing events, which increase in severity or change in nature during or as a consequence of use of a medicinal product in human clinical trials, will also be considered AEs.

Any medical condition or clinically significant laboratory abnormality with an onset date before the screening visit and not related to study procedures is considered to be pre-existing, and should be documented in the case report form.

Any AE (i.e., a new event or an exacerbation of a pre-existing condition) with an onset date after the screening visit up to the last day on study (including the follow-up, off study medication period of the study), should be recorded as an AE on the appropriate CRF page(s).

An AE does not include:

- Medical or surgical procedures (e.g. surgery, Endoscopy, tooth extraction, transfusion); the condition that leads to the procedure are an adverse event.
- Pre-existing diseases or conditions or laboratory abnormalities present or detected prior to the screening visit that does not worsen.
- Situations where an untoward medical occurrence has not occurred (e.g. hospitalization for elective surgery, social and/or convenience admissions).
- Overdose of either study drug or concomitant medication without any signs or symptoms unless the subject is hospitalized for observation.

***Assessment of Adverse Events***

All AEs will be assessed by the investigator and recorded on the appropriate CRF page, including the date of onset and resolution, severity, relationship to study drug or study procedures, outcome and action taken with study medication.

The relationship to study drug therapy or study procedures should be assessed using the following definitions:

**No**: Evidence exists that the adverse event has an etiology other than the study drug or study procedures (e.g. pre-existing condition, underlying disease, intercurrent illness, or concomitant medication).

**Yes**: A temporal relationship exists between the event onset and administration of the study drug or between the event and the study procedures. It cannot be readily explained by the subject's clinical state or concomitant therapies and, in the case of the study drug, appears with some degree of certainty to be related based on the known therapeutic and pharmacologic actions or adverse event profile of the study drug. In case of cessation or reduction of the dose, the event abates or resolves and reappears upon re-challenge. It should be emphasized that ineffective treatment should not be considered as causally related in the context of adverse event reporting.

These criteria in addition to good clinical judgment should be used as a guide for determining the causal assessment. If it is felt that the event is not related to study drug therapy, then an alternative explanation should be provided.

***Serious Adverse Events***

**A serious adverse event** (SAE) is defined as follows:

Any adverse drug experience occurring at any dose that results in any of the following outcomes:

- Death;
- Life-threatening situation (subject is at **immediate** risk of death);
- In-patient hospitalization or prolongation of existing hospitalization (excluding those for study therapy or placement of an indwelling catheter, unless associated with other serious events);
- Persistent or significant disability/incapacity;

- Congenital anomaly/birth defect in the offspring of a subject who received study drug;

Other: medically significant events that may not result in death, be immediately life-threatening, or require hospitalization, may be considered a SAE when, based upon appropriate medical judgment, they may jeopardize the Subject and may require medical or surgical intervention to prevent one of the outcomes listed in this definition.

Examples of such events are:

- Intensive treatment in an emergency room
- Blood dyscrasias or convulsions that do not result in hospitalization
- Development of drug dependency or drug abuse

The investigator should notify the Institutional Review Board (IRB) or Independent Ethics Committee (IEC) as soon as is practical, of serious events in writing where this is required by local regulatory authorities, and in accordance with the local institutional policy.

**References**


1. Buskila D, Neumann L. Assessing functional disability and health status of women with fibromyalgia: validation of a Hebrew version of the Fibromyalgia Impact Questionnaire. J Rheumatol. 1996; **23**(5): 903-6.

2. Peveler RC, Fairburn CG. Measurement of neurotic symptoms by self-report questionnaire: validity of the SCL-90R. Psychol Med. 1990; **20**(4): 873-9.

3. McHorney CA, Ware JE, Jr., Lu JF, Sherbourne CD. The MOS 36-item Short-Form Health Survey (SF-36): III. Tests of data quality, scaling assumptions, and reliability across diverse patient groups. Med Care. 1994; **32**(1): 40-66.

4. McHorney CA, Ware JE, Jr., Raczek AE. The MOS 36-Item Short-Form Health Survey (SF-36): II. Psychometric and clinical tests of validity in measuring physical and mental health constructs. Med Care. 1993; **31**(3): 247-63.

5. Neumann L, Berzak A, Buskila D. Measuring health status in Israeli patients with fibromyalgia syndrome and widespread pain and healthy individuals: utility of the short form 36-item health survey (SF-36). Semin Arthritis Rheum. 2000; **29**(6): 400-8.

6. Eriksson M, Lindstrom B. Validity of Antonovsky's sense of coherence scale: a systematic review. J Epidemiol Community Health. 2005; **59**(6): 460-6.

7. Sperber AD, Carmel S, Atzmon Y, Weisberg I, Shalit Y, Neumann L, et al. The sense of coherence index and the irritable bowel syndrome. A cross-sectional comparison among irritable bowel syndrome patients with and without coexisting fibromyalgia, irritable bowel syndrome non-patients, and controls. Scand J Gastroenterol. 1999; **34**(3): 259-63.

8. Muth CM, Radermacher P, Pittner A, Steinacker J, Schabana R, Hamich S, et al. Arterial blood gases during diving in elite apnea divers. Int J Sports Med. 2003; **24**(2): 104-7.
